# Organoids as a new model for improving regenerative medicine and cancer personalized therapy in renal diseases

**DOI:** 10.1038/s41419-019-1453-0

**Published:** 2019-02-27

**Authors:** Ludovica Grassi, Romina Alfonsi, Federica Francescangeli, Michele Signore, Maria Laura De Angelis, Antonio Addario, Manuela Costantini, Elisabetta Flex, Andrea Ciolfi, Simone Pizzi, Alessandro Bruselles, Matteo Pallocca, Giuseppe Simone, Mustapha Haoui, Mario Falchi, Michele Milella, Steno Sentinelli, Paola Di Matteo, Emilia Stellacci, Michele Gallucci, Giovanni Muto, Marco Tartaglia, Ruggero De Maria, Désirée Bonci

**Affiliations:** 1 IRCCS, Regina Elena National Cancer Institute, Rome, Italy; 2 Department of Oncology and Molecular Medicine, Istituto Superiore di Sanità, Rome, Italy; 3 Department of Internal Medicine and Medical Specialties, “La Sapienza” University, Rome, Italy; 4 RPPA Unit, Proteomics Area, Core Facilities, Istituto Superiore di Sanità, Rome, Italy; 5 Istituto di Patologia Generale Università Cattolica del Sacro Cuore, Largo Francesco Vito 1, 00168 Rome, Italy; 6 Oncological Urology Department, Regina Elena National Cancer Institute, Rome, Italy; 7 Department of Bioscience, Biotechnology and Biopharmaceutics, University of Bari, Bari, Italy; 8 Genetics and Rare Diseases Research Division, Ospedale Pediatrico Bambino Gesù, Rome, Italy; 9 National AIDS Center, Istituto Superiore di Sanità, Rome, Italy; 10 Section of Oncology, Department of Medicine, University of Verona School of Medicine, Verona, Italy; 11 Verona University, Hospital Trust, Verona, Italy; 12 Department of Urology, Humanitas University, Turin, Italy; 13 Scientific Vice-Direction, Fondazione Policlinico Universitario “A. Gemelli” - I.R.C.C.S. Largo Francesco Vito 1-8, 00168 Rome, Italy

## Abstract

The pressure towards innovation and creation of new model systems in regenerative medicine and cancer research has fostered the development of novel potential therapeutic applications. Kidney injuries provoke a high request of organ transplants making it the most demanding system in the field of regenerative medicine. Furthermore, renal cancer frequently threaten patients’ life and aggressive forms still remain difficult to treat. Ethical issues related to the use of embryonic stem cells, has fueled research on adult, patient-specific pluripotent stem cells as a model for discovery and therapeutic development, but to date, normal and cancerous renal experimental models are lacking. Several research groups are focusing on the development of organoid cultures. Since organoids mimic the original tissue architecture in vitro, they represent an excellent model for tissue engineering studies and cancer therapy testing. We established normal and tumor renal cell carcinoma organoids previously maintained in a heterogeneous multi-clone stem cell-like enriching medium. Starting from adult normal kidney specimens, we were able to isolate and propagate organoid 3D-structures composed of both differentiated and undifferentiated cells while expressing nephron specific markers. Furthermore, we were capable to establish organoids derived from cancer tissues although with a success rate inferior to that of their normal counterpart. Cancer cultures displayed epithelial and mesenchymal phenotype while retaining tumor specific markers. Of note, tumor organoids recapitulated neoplastic masses when orthotopically injected into immunocompromised mice. Our data suggest an innovative approach of long-term establishment of normal- and cancer-derived renal organoids obtained from cultures of fleshly dissociated adult tissues. Our results pave the way to organ replacement pioneering strategies as well as to new models for studying drug-induced nephrotoxicity and renal diseases. Along similar lines, deriving organoids from renal cancer patients opens unprecedented opportunities for generation of preclinical models aimed at improving therapeutic treatments.

## Introduction

Regenerative medicine research is based on the rapid advances in stem cell investigation, tissue-engineering and patient-derived models^1,2^. Understanding normal and cancer tissue organization may improve tissue engineering approaches as well as cancer drug discovery, since the regulation of developmental and regenerative processes of normal tissue share common traits, e.g., self-renewal, with the onset and spreading of cancer. Chronic kidney diseases are a major global health issue^3–6^. Since about 1.4 million patients, with an 8% of increase per year, are affected by renal diseases^7,8^, kidney transplantation has the highest request^9^. In addition drug nephrotoxicity represents a frequent side effect impacting on renal function^10,11^. Many efforts are devoted to improve stem-cell-based and tissue regenerative technologies. Ethical limitations foster the use of adult-patient-derived-pluripotent stem models including organoids which represent recent technological breakthrough^12^. Organoids seem suitably mimic original complex tissue organization^13–15^. The first evidence showed organoid cultures reproducing crypts of adult intestine^16^ followed by further innovative applications^17,18^. Organoid models raise expectations in tissue repair^19–23^ and cancer therapy testing^20,24–26^ research. Cancer-derived-organoids have been established from diverse organs^17,18,22,27–31^. Renal cancer (RCC) is one of the ten most common adult malignancies, accounting for approximately 3% of all adult tumors^32–34^ and distinguished in nineteen different subtypes^35^. The clear cell (cc) RCC is the most common subtype representing approximately 75%^33^. Surgery remains the only “curative” option for renal tumors with approximately one third of patients showing regional or distant metastases at diagnosis. Despite the high curative potential of surgery for localized masses, still one fourth of patients with localized RCC relapse in distant sites after surgery^36–39^. The prognosis is poor, with a current overall 5-year survival rate of 74%, decreasing to 53% for patients with loco regional disease (stage III) and dropping to 8% for patients with metastatic disease (stage IV)^40^. To date, organoid models for renal cancer are unsatisfying. The establishment of patient-derived models faithfully reproducing original tumors are essential to investigate molecular mechanism, identify new diagnostic, prognostic biomarkers and personalized patient treatments. Improving in vitro models may have several advantage compared to in vivo systems including compatibility with high throughput assays, such as genomics, proteomics and drug screenings and above all related ethical issues. Since organoids preserve tumor heterogeneity, they mimic the original patient tissue better than cell lines and other in vitro patient-derived models^41^. The proven reliability and in vitro–in vivo dual usability, encourage organoid model use for studying tumorigenesis and driver mutations^42,43^. Complex platforms matching results of genome sequencing and other “omics” coupled to drug screening on patient-derived organoids were devised for improved personalized medical approaches^44,45^. Our innovative results pave the way to the exploitation of normal and cancer renal organoid cultures and may help to improve tissue engineering approach, regeneration therapies and nephrotoxicity studies as well as for ccRCC research.

## Materials and methods

### Organoid cultures

Tissue samples were collected at Regina Elena” National Cancer Institute (Rome), in accordance with procedures approved by ethical committee. Surgical specimens were washed several times with DPBS (Dulbecco’s phosphate-buffered saline, Invitrogen) additioned with metronidazole at 20% (Braun) and Antibiotic-Antimycotic at 4% (Sigma-Aldrich). Tissue dissociation was performed first by mechanical dissociation with sterile scissor followed by enzymatic digestion in Dulbecco’s Modified Eagle Medium (DMEM) High Glucose with L-Glutamine (Sigma-Aldrich), additioned with ialuronidase IV (2 μl/ml Sigma-Aldrich) and collagenase II (10 μl/ml Invitrogen) for 45 min at 37 °C. The resulting cell suspension was then plated in non-tissue-culture-treated flasks in a serum-free, stem cell enriching medium having the following composition: insulin (50 μg/ml, Sigma-Aldrich), apo-transferrin (100 μg/ml, Sigma-Aldrich), putrescine (10 μg/ml, Sigma-Aldrich), sodium selenite (0.03 mM, Sigma-Aldrich), glucose (0.6%, Sigma-Aldrich), HEPES (5 mM Sigma-Aldrich), sodium bicarbonate (0.1%, Sigma-Aldrich), Bovine Albumin Cohn Fraction V (BSA) (0.4%, MP Biomedical), glutaMAX (×1, Gibco-Invitrogen), Penicillin-Streptomycin (×1, Sigma-Aldrich), EGF (Epidermal Growth Factor, 20 μg/ml, PeproTech) and bFGF (basic Fibroblast Growth Factor, 10 μg/ml, PeproTech) dissolved in DMEM–F12 medium (Gibco-Invitrogen)^46^. After 72 h incubation in the stem cell enriching medium, the cells and aggregates obtained where gently dissociated with Tryple Express (Thermo Fisher), embedded in Growth factor reduced Matrigel (Corning), and cultured in basic organoid medium composed by: HEPES (10 mM, Sigma-Aldrich), GlutaMAX (×1, Gibco-Invitrogen), B27 (×1, Invitrogen) N-acetyl-L-cysteine (1 mM, Sigma-Aldrich) A83-01 (500 nM, Tocris), EGF) (20 μg/ml, PeproTech), bFGF (10 μg/ml, PeproTech), Rho-associated protein kinase (ROCK) inhibitor (10 nM, Selleckchem) and Penicillin-Streptomycin (×1, Sigma-Aldrich) dissolved in Advanced DMEM/F12 medium (Invitrogen). Medium was refreshed once per week and cultures were passaged at an average dilution factor of 1.3–1.6, once per week.

### Organoid culture treatments

Normal organoids were cultivated with 20 μM or 100 μM Cisplatin for 48 hours (hrs) and 72 hrs for cytotoxicity evaluation. Cancer and normal parental organoids were maintained in culture with 10 nM and 10 μM of Sunitinib and Tensirolimus respectively for 72 hrs following manual instruction (Selleck, www.selleckchem.com). Cancer organoids and their normal counterparts were treated with SU11274 10 µM, FORETINIB 10 μM, LENVATINIB in combination with EVEROLIMUS (both at final concentration of 10 µM) and Cabozantinib 10 μM following manual instruction (Selleck, www.selleckchem.com).

### RNA sequencing, DNA mutation analysis, statistical analysis, and short tandem repeat evaluation

For whole exome and mRNA sequencing (WES and RNAseq, respectively), we took advantage of the DNA Link Sequencing service (www.dnalinkseqlab.com). Briefly, genomic DNA was processed for exome-targeted library enrichment using the Twist Human Core Exome Kit (Twist Bioscience, San Francisco, CA, USA) and the library for RNAseq was prepared by TruSeq Stranded mRNA Library Prep Kit (Illumina, San Diego, California, USA). For WES sequencing details, analysis and quality controls, see Supplementary material. mRNA sequencing was performed on a NovaSeq 6000 instrument (Illumina, San Diego, California, USA) and, for analysis of raw sequencing data, we employed custom pipelines. RNAseq fastq files underwent quality check and processed to obtain Transcripts Per Million (TPM), normalized expression data, using Kallisto^47^. Short Tandem Repeat Analysis for sample Identification in organoid and parental derivative tissues were performed with AmpFℓSTR™ Identifiler™ PCR Amplification Kit (Catalog Number 4322288 Thermofisher Scientific). Mutation scanning of the specific variants selected among frequently published (Saeed K. et al.^48^ and TCGA genome ATLAS^49^) was performed by Sanger sequencing using an ABI Prism 3500 Genetic Analyzers (Applied Biosystems) and the ABI BigDye Terminator Sequencing Kit V.3.1 (Applied Biosystems). Primer pairs designed to amplify a specific coding regions of PBMR1, TSC2, VHL, KDM5C, SETD2 and PIK3C2A genes are listed in online Supplementary Information. All Prep DNA/RNA Mini kit (Qiagen) was used for RNA and DNA preparations. Statistical comparisons and graphical representation of RNAseq expression levels as well as WES variants data were performed by custom “R” scripts, available upon request. In details, we used “R” v3.5 (https://www.r-project.org/) and RStudio v1.1 (https://www.rstudio.com/) with the following ‘R’ packages: plyr, tidyverse, ggsignif, ggbeeswarm, data.table, coin, car and exactRankTests. In details, we converted TPM data to base two logarithm and used Lilliefors and Shapiro-Wilk normality tests to assess the normal distribution. In the statistical analysis of selected genes [the results were deviated significantly from null hypothesis (i.e., normal distribution)] we opted for non-parametric statistics, i.e Wilcoxon Rank Sum or Signed Rank, depending on the number of data points in each group. Exact p values were calculated and control of false discovery rate was performed as per Benjamini Y. and Hochberg Y [Controlling the false discovery rate: a practical and powerful approach to multiple testing. Journal of the Royal Statistical Society Series B (Methodological), 1995]. Statistical significance is reported on plots using the following notation: *P < 0.05; **P < 0.01; ***P < 0.001.

For Supplementary Information, materials and methods see Supplementary Information on line.

## Results

### Establishment of healthy kidney organoid cultures

**Fig. 1:**
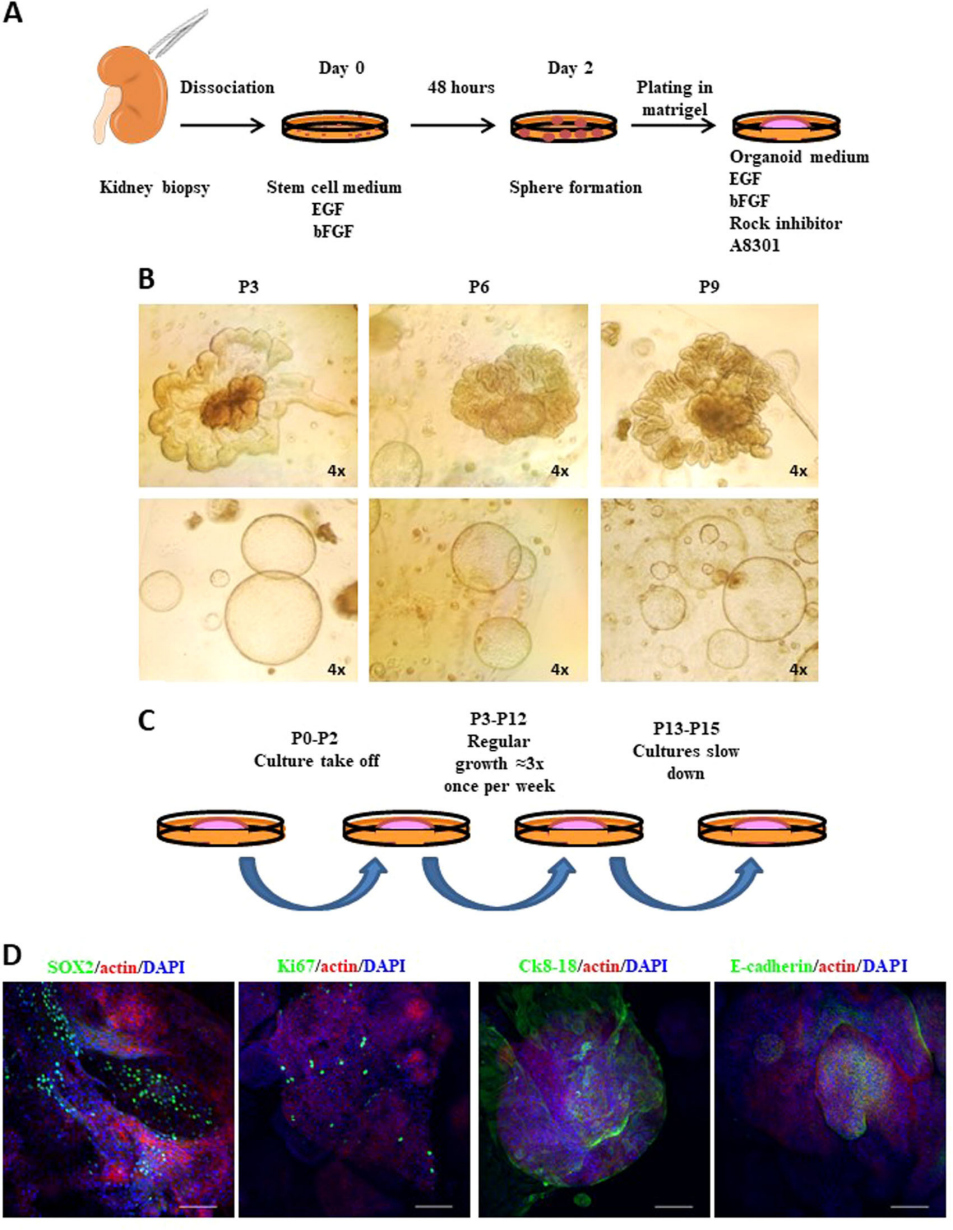
Establishment of normal kidney organoid cultures. **a** Schematic drawing of organoid culture isolation. Fresh tissues are dissociated, cultured for 72 h in the stem-cell-enriching medium and finally plated in Matrigel into organoid medium. **b** Representative images of normal kidney organoid cultures at passage (P) 3, 6, and 9. Two different populations can be distinguished: well structured (top panels) and round-shaped aggregates (lower panels). Microscope magnification ×4. **c** Scheme of the cultures trends over passages. **d** Representative confocal images of immunofluorescence stainings for SOX2, Ki67, Ck8–18, E-cadherin (green) and actin (red). Nuclei are stained with DAPI (blue). Microscope magnification ×20. Scale bars 100 μm

Thirteen surgery specimens obtained from normal tissues of neoplastic patients (clear cell, hybrid cromophobe, renal urothelial, renal neuroendocrine diseases) who underwent total nephrectomy were used for culture condition optimization. Specimens explanted under direction of a pathologist were mechanically and enzymatically dissociated and maintained in a serum-free medium^50–54^. Cellular suspensions were grown with typical stem-cell-selective growth factors, i.e., Epidermal Growth factor (EFG) and basic Fibroblast Growth Factor (bFGF)^46^. This medium favors undifferentiated cell enrichment while depleting the culture from hematopoietic contaminant populations (Supplementary Fig. 1). After 72 hours (hrs or h), suspensions were plated in a specific medium composed by low-density Matrigel supplemented with EGF, bFGF, Rho kinase (ROCK) inhibitor and A8301, a selective inhibitor of the Transforming Growth Factor β type I Receptor (TGF-β), the Anaplatistic lymphoma kinase 4 and 7 (ALK4 and ALK7)^16,17,28^, (Fig. 1a and Supplementary Table 1). The organoid formation was clearly visible by microscope evaluation within a couple of passages (corresponding to two weeks of culture). The culture establishment success rate was very high involving 100% of samples. Interestingly, we could observe two different organoid types: a very organized and structured category and a rounded shaped cystic form group (Fig. 1b). Normal organoids displayed regular growth (1.3 once per week) and could be propagated for a long period (Fig. 1c). Among samples, only one culture ceased to proliferate after passage 9 (9 weeks from plating in Matrigel), while the others required passaging up to the fifteenth week from initial plating in Matrigel. Short Tandem Repeat Analysis for derivation identity evaluation comparing organoid and parental tissues was performed. Results demonstrated parental derivative origin of organoid cultures (Supplementary Fig. 2A and Supplementary File 1).

### Characterization of normal kidney organoids

**Fig. 2:**
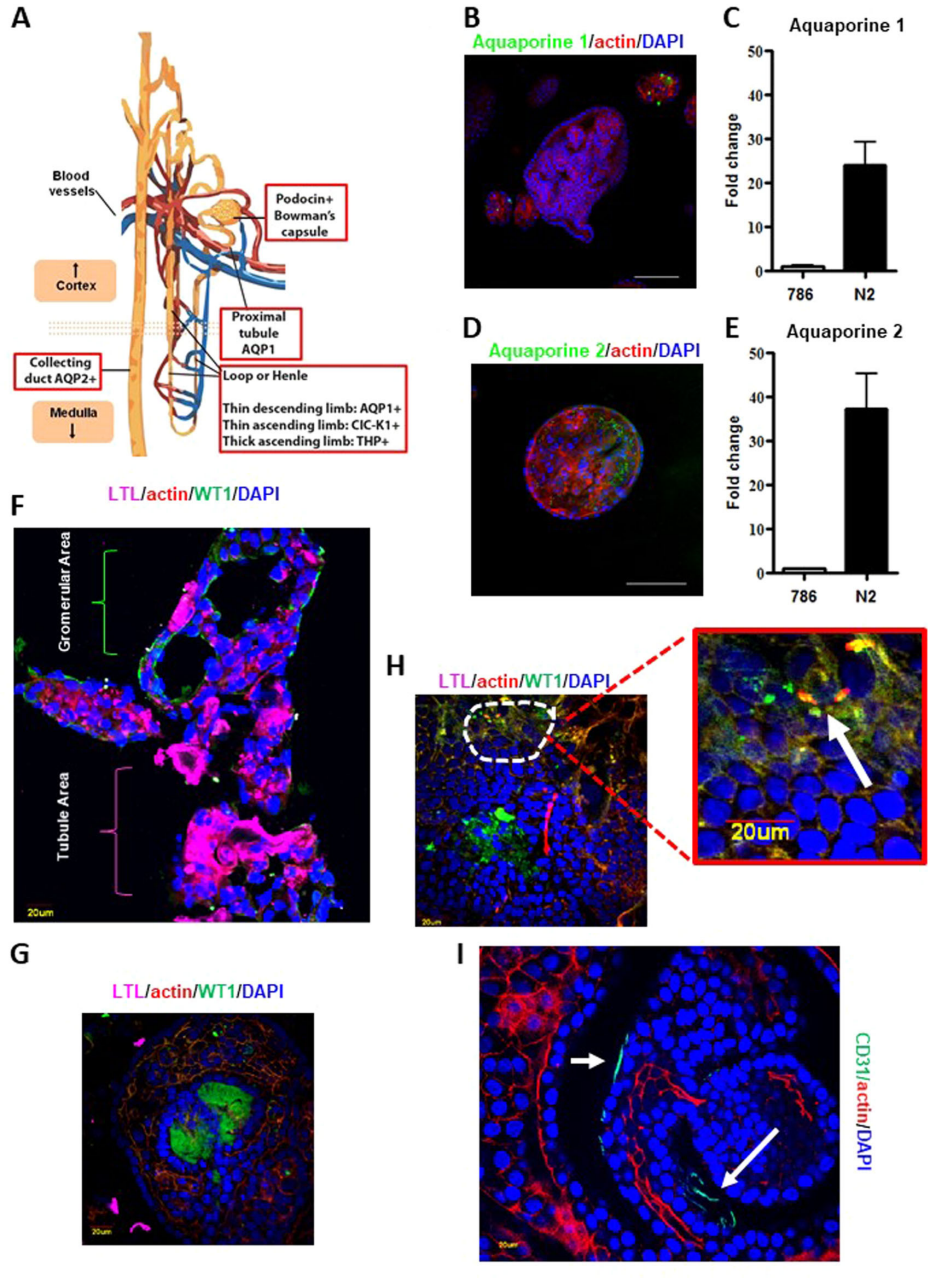
Characterization of normal kidney organoid cultures. **a** Cartoon of renal markers specific for each region of the nephron. **b** Representative confocal images stained for Aquaporin 1 (green) and Actin (red). Nuclei are stained with DAPI (blue). Microscope magnification ×20. Scale bars 100 μm. **c** mRNA expression assessed by qPCR of Aquaporine 1 (Aqp1) in the same normal kidney organoid culture. “786” commercial RCC cell line is used as negative control. Results are shown as a mean ± SD of at least three independent experiments. **d** Representative confocal images stained for Aquaporin 2 (green) and Actin (red). Nuclei are stained with DAPI (blue). Microscope magnification ×20. Scale bars 100 μm. **e** mRNA expression assessed by qPCR of Aquaporine 2 in the same normal kidney organoid culture. “786” RCC line is used as negative control. Results are shown as a mean ± SD of at least three independent experiments. Immunofluorescence images are representative of at least five independent experiments. **f** Organoids were included in cryomatrix (OCT), cutted in 8 μM slides and stained for WT1, LTL, Actin and DAPI markers. Representative confocal images were reported. **g**–**h** Fresh fixed and permeabilized organoids evaluated for LTL, WT1, Actin and DAPI markers by immunofluorescence assay. Representative confocal images were reported. **i** Fresh fixed and permeabilized organoids evaluated for CD-31, Actin and DAPI markers by immunofluorescence assay. Representative confocal images were reported

In order to characterized organoids in their preserved 3D-structure, we analyzed spheroids by immunofluorescence (Supplementary movie 1–2). Organoid cultures were enriched of a SOX2-expressing population, mainly localized in the core of structures and indicative of the presence of stem/progenitor cells responsible for self-renewal and propagation (Fig. 1d). Immunofluorescence analysis also highlighted the presence, mostly at the periphery of 3D-cultures, of a differentiated population expressing CK8-18 and E-cadherin, two typical markers of the urinary tract epithelium. In the organized aggregates, we detected cells positive for the proliferation marker Ki67, underlining the presence of actively dividing cells (Fig. 1d). Since the presence of human renal adult-stem-cells remains questionable^55–59^, we hypothesized the existence in organoids of (committed) stem/progenitor cells underlying the maintenance and propagation of cultures. The kidney epithelium cells are podocin positive in the Bowman capsule; aquaporin 1 positive (AQP1+) in the proximal tubule and the thin descending limb of the hemle loop; ClC-K1 (an ionic channel) positive (ClC-K1+) in the thin ascending limb; Tamm–Horsfall glycoprotein positive (THP+) in the thick ascending limb, and aquaporin 2 positive (AQP2+) in the collecting duct (Fig. 2a). Therefore, several specific markers for each nephron region were investigated by immunofluorescence and real time PCR (qPCR). Specific marker expression levels varied from case to case (Supplementary Fig. 2B). Indeed, a fraction of the analyzed samples showed high levels of aquaporin 2 and aquaporin 1, the latter being almost selectively expressed in small-sized organoids (Figs. 2b–e). To compare organoids and parental tissues five fresh normal specimens were specifically collected from medulla and cortex area (two and three respectively) under direction of a pathologist (Supplementary Fig. 3A). Transcriptome analysis comparing the samples was run. In detail, RNA-seq-trascriptome analysis was performed pointing attention to several selective genes for different kidney areas such as recently published^60^. Expression profiles were reported after two-way-hierarchical-cluster analysis in heat-map (Supplementary Fig. 3B). Statistical evaluation of data showed many genes expressed both in organoid cultures and parental tissues while several transcripts were significantly associated to organoids or tissues (Supplementary Figs. 3C and 4). Of note, two-way analysis showed a good clusterization of cortex vs medulla organoids with a marked different expression of a sub-group of transcripts (Supplementary Fig. 5). As first morphological approach, organoids were included in frozen tissue matrix (OCT) and evaluated by haematossylin and Eosin (H&E) staining and immunofluorescence. H&E staining seamed resembled tubule structures in organoids such as in tissue counterpart (Supplementary Fig. 6). WT1 (Wilms’ Tumor) and LTL (lotus tetragonolobus lectin) proteins are frequently associated to glomerular and tubule areas respectively^14,15,61^ (Supplementary Fig. 7A). To further investigate organoids obtained from renal cortical or medullary parts we analyzed WT1 and LTL pattern expressions in crio-matrix preserved and fresh fixed structures by immunofluorescence. Organoids showed areas of gromerular and tubule structures (Figs. 2f, g and Supplementary Fig. 7B-C; Supplementary Fig. 7C represents the zoom of Fig. 2f). However, we did not notice expression differences in term of WT1 and LTL localizations and levels when compared to cortex and medulla area derivation indicating organoids showed both traits of tissue districts. Intriguingly, several areas retained WT1 and LTL co-expressing cells (Fig. 2h and Supplementary Fig. 7D). WT1 gene may be associated with stem cell properties^14,15,61^ while LTL co-expression may suggest the enrichment of stem-progenitor cells able to produce different anatomical portions. Despite evidence show the cell of origin determines the molecular expression scenario in kidney cancer^62^ further studies are required to elucidate this in renal organoid settings. Organoids also demonstrated complex heterogeneous structure mirroring angiogenic network such as parental tissues by RNA-Seq analysis (Supplementary Fig. 8A). CD-31 antigen expression and localization confirmed that organoids mimic angiogenesis architectures as by immunofluorescence assay (Fig. 2i and Supplementary Fig. 8B). Angiogenesis reproduction in organoid structures foster expectation, however, requires further functional studies. While organoids may fail as model for studying microenvironment and immune system, recent studies showed new frontiers of co-culture applications of organoids and lymphocytes^63,64^. In this direction, our data may offer new attractive models for renal disease investigation.

### Normal kidney organoids as cytotoxicity assay

**Fig. 3:**
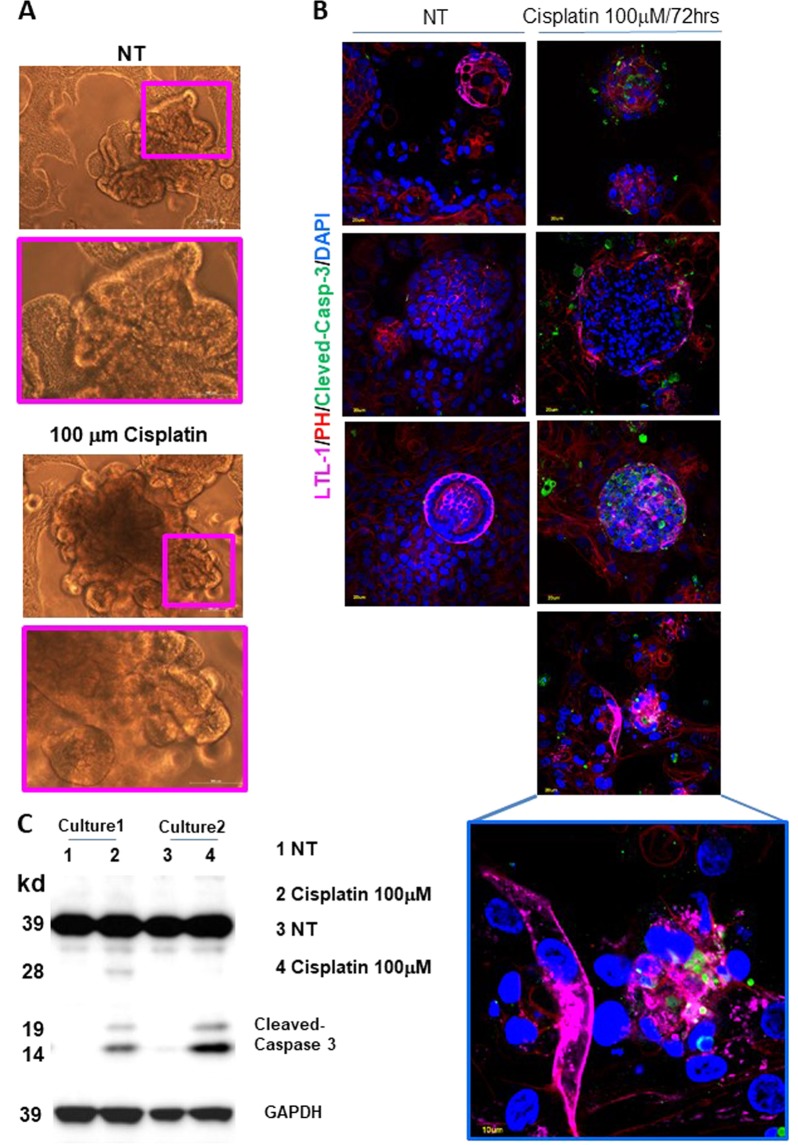
Normal kidney organoids as cisplatin cytotoxicity assay. **a** Representative phase contrast images of organoid cultures after 72 h of 100 μM Cisplatin exposition. Organoid untreated cultures (NT) were used as control. **b** Representative images of 100 μM cisplatin treated and untreated (NT) organoid cultures evaluated after 72 h for LTL, Cleaved-Caspase 3, Actin and DAPI markers by immunofluorescence stainings. **c** Cleaved-Caspase 3 expression evaluated in 100 μM cisplatin treated and untreated (NT) organoid cultures after 72 h by Western blotting. Representative images of two organoids cultures were reported. GAPDH protein was used as internal control

Normal organoids may represent intriguing models for drug cytotoxicity estimation. Cisplatin, a widely used cancer chemotherapeutic agent, may have nephrotoxic side effect. In fact, it seems to induce apoptosis in proximal tubules and to impair migration capacity of endothelial cells^61,65,66^. To assess the value of our models as cytotoxicity test, organoids were exposed to different cisplatin doses for 48 and 72 h. Cisplatin did not affect organoid structures at both doses after 48 h of treatment as by phase contrast (PH) images and by absence of Cleaved-Caspase-3 activation by Western blotting (Supplementary Fig. 9 and 10A). Endothelial cells seemed not to be affected, CD-31 expression level did not change by Western Blotting (Supplementary Fig. 10A) while modified its localization (Supplementary Fig. 10B). Of note, normal organoids showed a consistent Cleaved-Caspase 3 activation and apoptosis induction after 72 h at 100 μM dose by Western blotting, Tunel assay and immunofluorescence (Fig. 3 and Supplementary Fig. 11). Organoid architecture was not affected by drug exposition as by PH images (Fig. 3a) suggesting that only a subtype of renal cells were sensitive to cisplatin. Thus, we evaluate the co-expression of Cleaved-Caspase 3 with LTL and WT1 antigens and we found that only tubule cells suffered after cisplatin exposition (Fig. 3b and Supplementary Fig. 12). Data showed that organoid cultures may be used as 3 D complex and variegated model for drug side effect testing.

### Establishment of ccRCC organoid cultures

**Fig. 4:**
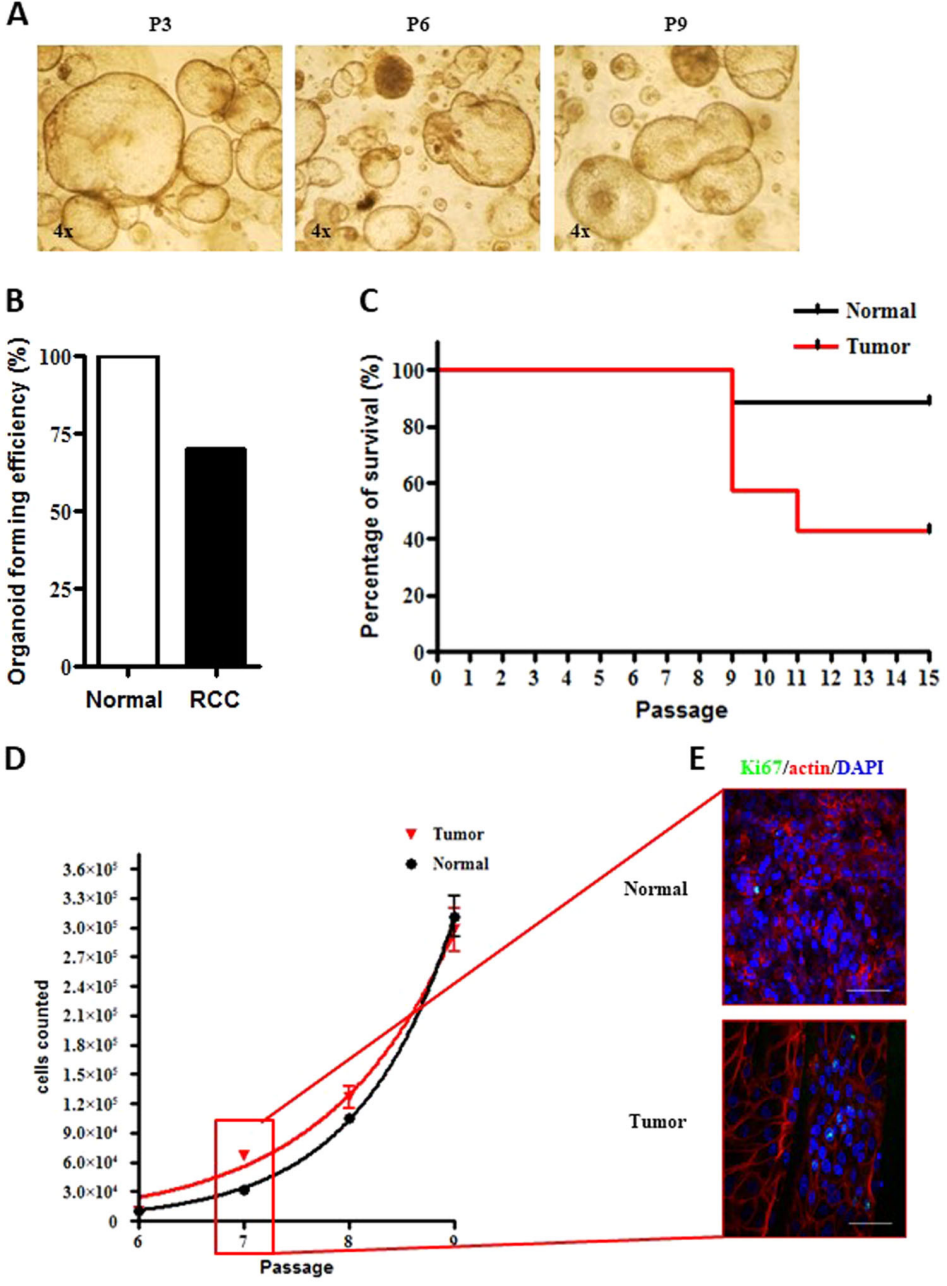
Establishment of RCC organoid cultures. **a** Representative images of RCC kidney organoid cultures at passage (P) 3, 6 and 9. Microscope magnification ×4. **b** Percentage of organoid culture formation efficiency starting from normal and RCC fresh tissues. **c** Kaplan-Meier plot of culture persistence over passages of normal (black) and RCC (red) organoids. **d** Illustration of the number of cells counted in normal (black) and RCC (red) organoids from passages 6 to 9. Results are expressed as mean ± SD of three independent cultures. **e** Representative confocal microscope images of normal (upper panel) and tumor (lower panel) organoids stained for Ki67 (green) and actin (red). Nuclei are stained with DAPI (blue). Microscope magnification ×60. Scale bars 50 μm. Immunofluorescence images are representative of at least six independent experiments

We enrolled  fifteen clear cell renal cancer (ccRCC) patients who underwent total or partial nephrectomy (Supplementary Table 2). Freshly explanted samples of both tumor and normal counterparts were collected under the guide of a pathologist. Following previously described protocol, organoid structures were obtained within a couple of passages (i.e., 2 weeks) from the first plating in Matrigel (Fig. 1a). Differently from normal cultures tumor samples produced only rounded shaped population, in line with literature^16,17,28^ (Fig. 4a). The success rate of tumor culture establishment was inferior to that of the normal organoids. In fact, only 10 out of 15 samples generated tumor organoids, while all normal parental tissues gave rise to 3D-structures in vitro (Fig. 4b). Success rate difference may be due to severe necrosis that usually affects renal tumor tissues. Furthermore undifferentiated tumor populations while lose contact inhibition and differentiation capacity may acquire invasive properties and fall to create 3D-structures. Interestingly, tumor organoid cultures compared to normal ones demonstrated an increased growth rate between passage 5–9, with a two-fold greater duplication time during the exponential phase (Figs. 4c–e). However, while normal cultures were able to preserve their ability to form organoid structures after several passages, the tumor ones showed decreased capacity of long-term colony/structure formation (Fig. 4b). Indeed, the majority of the tumor cultures underwent a resting phase at passage 10. Notably, three samples derived from Stage 4 tumors were propagated up to passage 15 (Fig. 4c). Short Tandem Repeat Analysis for sample identification in tumor organoid and parental derivative tissues was performed. Analysis demonstrated parental derivation of cancer organoid cultures (Supplementary Table 3 and Supplementary File 2). Furthermore, cancer genomic patient representation was evaluated in three couples of tumor organoids vs parental tissues by WES-DNA-exome-sequencing. Allele frequency of wild type and variant genes significantly demonstrated a multiclonal representative capacity of tumor organoid cultures when compared to parental cancer tissue. In fact, only about 5% of allele setting resulted different among tumor organoids and tissues (Supplementary Fig. 14A-B and Supplementary File 3). Since data demonstrated wild type/variant multi-clone proportion strong maintenance among tumor tissues and organoids, our cultures may be good patient mirroring models (Supplementary File 3). Three normal organoid cultures were additionally compared to their tissue counterparts (Supplementary Table 4). Wild type/variant multi-clone proportions were highly preserved among normal tissues and derivative organoids. In addition, two paired normal and tumor tissues, chosen among the three above, together with their organoid counterparts were analyzed for most renal cancer frequent mutations^67–69^. In detail, tumor vs normal tissue mutation analysis was compared to tumor vs normal organoids genomic setting (Supplementary Fig. 14C and Supplementary file 4). Data showed that a panel of mutations frequently associated to ccRCC were shared among both analysis suggesting that tumor organoids can represent mutational patterns of derivative cancer over normal counterparts (Supplementary Fig. 14C-D and Supplementary File 4). These results suggest organoids suitably reproduced derivative tissues offering intriguing new frontiers of investigation. In order to evaluate the tissue bio-banking potentiality we tried to obtain organoid cultures from long-term stored samples. We selected eight cryopreserved tumor tissues (Supplementary Table 5) that had been maintained in serum and liquid nitrogen condition. After thawing and washing with buffer saline solution, samples were dissociated following the protocol. Out of eight tested samples, four produced organoid cultures. The feasibility of cryopreserved tumor samples, may pose the bases for improving overtime management of recurrent and advanced patients. Despite our technical approach has room for improvement, these findings hold unprecedented relevance since we demonstrated the using of long-term-storage patient material. This may be useful during therapy monitoring approach for appropriate drug choice. Thus, renal organoids may offer new tools for patient material propagation and therapy personalization in recurrent or therapy resistant patients.

### Characterization of ccRCC organoids

**Fig. 5:**
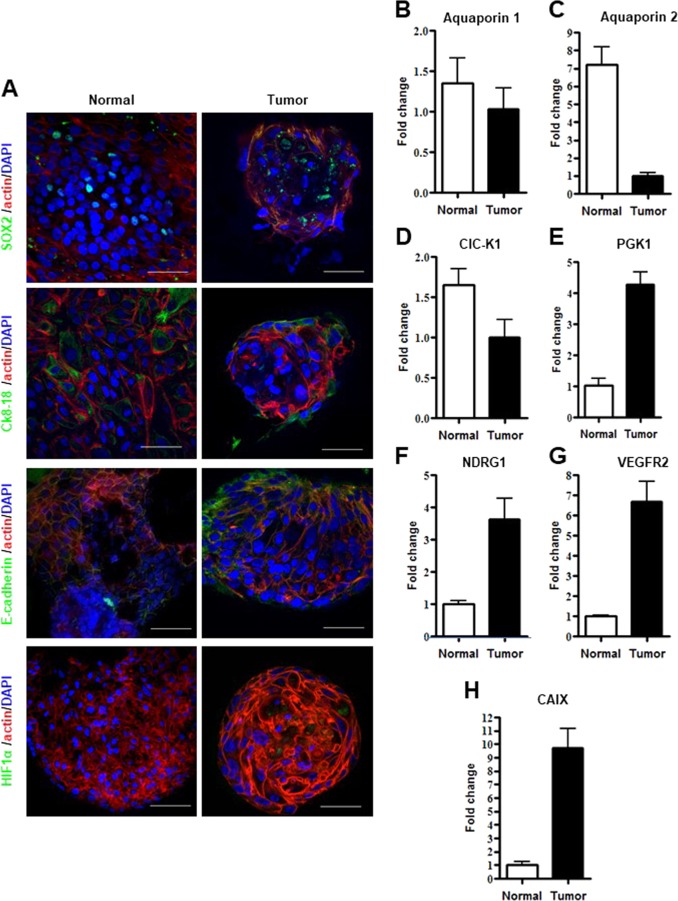
Characterization of RCC organoid cultures. **a** Representative confocal microscope images of normal (left panel) and tumor organoid (right panel) stained for SOX2, Ck8-18, E-cadherin and HIF1α (green), respectively. Nuclei are stained with DAPI (blue) and Actin staining is pseudo-colored in red. Microscope magnification ×60. Scale bars 50 μm. **b**–**h** mRNA expression, assessed by qPCR, of Aquaporine 1, Aquaporine 2, ClC-K1, PGK1, NDRG1, VEGFR2, CAIX in normal kidney and RCC organoid cultures. Results are shown as a mean ± SD of at least three independent experiments. Immunofluorescence images are representative of at least six independent experiments

ccRCC 3D-structures were characterized by immunofluorescence (Supplementary movie 3). Interestingly, ccRCC aggregates showed a different organization as compared to normal organoids when analyzed by confocal microscopy. In details, tumor cultures were composed of loosely organized three-dimensional structures of different sizes and composed by cells retaining big, aberrant nuclei (Supplementary Fig. 13A). Nonetheless, similarly to normal organoids, detection of a SOX2 positive population as well as of CK8-18 positive cells at the periphery of spheroid structures, pinpointed the presence of stem-like and differentiated compartments, respectively (Fig. 5a). Intriguingly, E-cadherin was strongly polarized in cancer organoids, suggestive of epithelial-mesenchymal transition state and aggressiveness (Fig. 5a), in line with reverse phase protein array (RPPA) (Supplementary Fig. 13B) and FACS analysis in normal (Supplementary Fig. 1) and ccRCC cultures previously published^46^. To compare tumor and normal parental cultures several additional markers were evaluated by real time PCR. We found Aquaporin 1 and 2 and CLC-K1 to be highly and modestly expressed in normal and tumor organoid cultures respectively (Figs. 5b–d). Since HIF1α is strictly associated to renal cancer and specifically correlated with tumor progression^70^, we evaluated its expression level in our cultures. HIF1α immunofluorescence analysis confirmed its expression in cancer but not in normal control organoids (Fig. 5a). We then investigated the expression levels of four HIF1α target genes: the Phosphoglycerate kinase 1(PGK1), the N-Myc Downstream Regulated 1 (NDRG1), the Vascular Endothelial Growth Factor Receptor 2 (VEGFR2) and the Carbonic Anidrase IX. All the aforementioned genes are markers of ccRCC and resulted overexpressed in tumor samples and almost absent in normal cultures (Figs. 5f–h). These findings reinforce our claim that we isolated pure normal and tumor organoid cultures.

### ccRCC organoids as drug testing

In order to evaluate organoid technology in drug testing we treated four ccRCC cultures and their normal counterparts with conventional targeted therapy, Sunitinib and Tensirolimus. Organoids were treated with different doses and analyzed after 72 h. mTOR S2448, VEGFR2 Y996, pERK T202/Y204 gene expression levels were evaluated as driver targeted therapy genes. All normal cultures were not affected at proliferative and driver targeted therapy gene levels by PH images (data not shown) and by Western blotting (Supplementary Fig. 15). Similarly, drugs did not impact on mTOR S2448, VEGFR2 Y996, pERK T202/Y204 and Cyclin D1 expression levels in tumor counterparts while Tensirolimus reduced cancer organoid capacity formation (Supplementary Fig. 15). We enlarged evaluation of cancer organoids as drug testing model exploring the activity of on-going trial drugs based on multi-kinases inhibition i.e SU11274, Foretinib, Cabozantinib and Levantinib in combination with Everolimus. Normal and tumor organoids isolated from two selected patients were characterized as mutational patterns by Sanger sequencing looking at the most frequent lesions recently associated at therapy in renal cancer^48^ (Supplementary Fig. 16–18). All sequences resulted wild type with the exception of a frameshift mutation in VHL gene [c.242 delC ins TG (p.Pro81Leu*50)] in one patient and retained in both tumor tissue and organoids. In the specific residue was identified the variants c.242 C > A (Pro81 Glu), c.242 delC (Pro81 fs*78) and c.242_243 insG (p.Arg82 fs*50) as reported in COSMIC database (Supplementary Fig. 18A and See Supplementary information). Normal and tumor organoids isolated from the two patients were treated with SU11274, Foretinib, Cabozantinib and Levantinib in combination with Everolimus, for 72 h. Normal cultures did not show any kind of toxicity as by PH analysis and Western blotting (Supplementary Figs. 16–18). pAKT S437 and pERK T202/Y204 were used as driver targeted genes of therapy efficacy. Cleaved-Caspase 3 level was evaluated for apoptosis induction by Western blotting. ccRCC organoids were affected by Foretinib and SU11274 while pAKT S437 and pERK T202/Y204 expressions were reduced in all the treatments (Supplementary Figs. 17–18) as by PH images and Western blotting. Of note, only Foretinib caused consistently Cleaved-Caspase 3 activation as by Western blotting (Supplementary Fig. 18B-C). Data suggest ccRCC organoids as new approach for therapy decision-making. Organoids may fail as model for studying immunotherapy and anti-angiogenesis drugs, however, new encouraging evidence suggest co-cultures of tumor organoid and parental lymphocytes as innovative approach^63,64^. In this direction we wanted to speculate the potentiality of our systems evaluating several antigens associated with tumor immune-surveillance. Since RANKL and IL-6, ccRCC previously published antigens^46^, were strongly correlated with immunotherapy resistance^71–73^, we run additional stainings for PD-L1 and PD-L2 in our ccRCC already reported^46^ heterogeneous cohort by RPPA. The new stainings demonstrated that our pre-undifferentiated enriched RCC-populations expressed PD-L1 and PD-L2 at different levels (Supplementary Fig. 19). Blue line was used as arbitrary high (Pink triangles) and low (Black circles) PD-L1 levels for underlining co-expressions or relative exclusions. Cases separating for high and low PD-L1 levels retained concordant expression of IL-6 while showed non-correlated expression of RANKL and PD-L2 (Supplementary Fig. 19). Since we produced tumor organoids from pre-enriched populations the expression of markers associated to immune-surveillance may suggest the future development of co-cultures for evaluating immunotherapy response.

### In vivo propagation of organoids

**Fig. 6:**
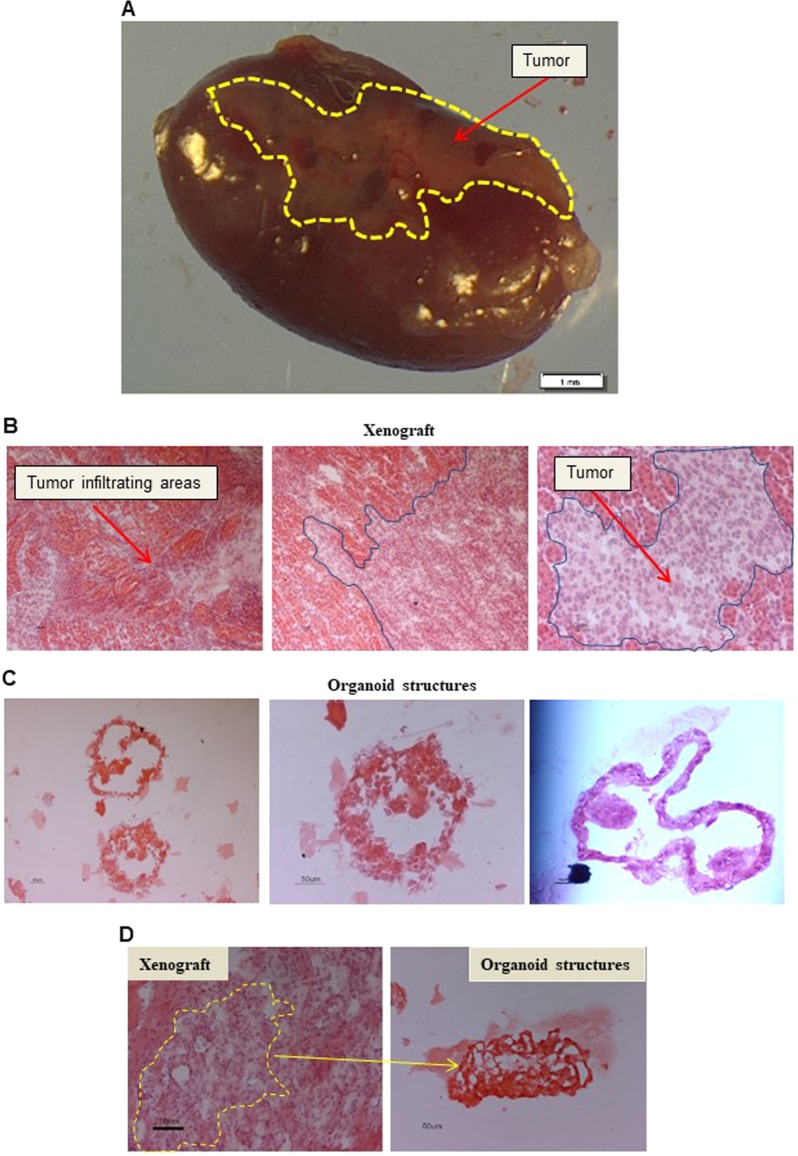
Organoids in vivo propagation. **a** Representative stereomicroscopic image of the tumor mass (Tumor, red arrow) excised 120 days after injection of a RCC organoid culture, under the renal capsule of immunocompromised mice. The cancer mass is highlighted by a yellow line. Scale bars 1 mm. **b** Representative H&E images of a xenograft derived from a G4 ccRCC organoid culture. Blue lines and red arrows indicate human tumor invading the mouse parenchyma. Microscope magnification ×10 (left and central panels) and 20 (right panel) (**c**) H&E staining of the original matrigel organoid culture included in OCT and cut in 5 μm slides for staining. Microscope magnification x20 Scale bars 50 μm. **d** H&E staining of the original matrigel organoid cultures and xenograft tumor masses included in OCT, cut in 5 μm slides for staining. Microscope magnification x20 Scale bars 50 μm

To investigate tumor propagating properties of cancer organoid cultures, we injected them under renal capsule of immunocompromised mice. Intact, non-dissociated 3D-structures were inoculated with Matrigel. Implanted organoids showed exceptional engraftment rates, with 3 cultures out of 4 growing (approximately 75%) (Fig. 6a and Supplementary Table 6). Haematoxylin and Eosin staining of explanted tumors confirmed their elevated capacity to invade the renal parenchyma (Fig. 6b). Haematoxylin and Eosin staining of tumor organoid cultures confirmed their elevated disorganized structures both in vitro and in vivo (Fig. 6c, d). These findings show that cancer organoid cultures retain tumor-propagating populations and can be maintained successfully both in vitro and in vivo. This model represents an additional patient-derived preclinical model with diverse, potentially useful applications.

## Discussion

Here, we describe the establishment and characterization of normal kidney and ccRCC organoid cultures derived from freshly explanted as well as from frozen patients’ tissues. Thus far, human normal kidney organoids have only been obtained from pluripotent embryonic stem cells^13,15,61,66,74,75^. Normal kidney organoid cultures hold an unprecedented potential of applicability in different fields such as disease modeling, organ replacement therapies, nephrotoxicity tests and developmental studies. They also provide normal controls for oncological studies. For drug screenings and nephrotoxicity studies, adult stem cell-derived organoids may represent the best option since they can be obtained from individual patients and they could be more physiological than embryonic and reprogramming-induced stem cell derived organoids. We optimized a new organoid model using adult stem cell multi-clonal populations. Organoid structures recapitulated the heterogeneity of tissue of origin and reproduced drug response, metabolism or toxic effects mirroring patient. For therapeutic purposes in tissue regeneration, adult kidney stem cell-derived organoids may represent a new source. Adjusting the composition of growth factors in the medium, may be possible to direct stem cell differentiation towards a specific fate^76^. This would allow re-growth of specific segments of nephron or selective part of renal organ. Importantly, organoid technology may be implemented for transplantation of damaged renal segment^77^. Nephrotoxicity studies, which would require reproduction of whole kidney, may benefit of organoids as drug-testing. CD-31 expression in our organoid system fosters expectations of innovative applications requiring angiogenesis compartment. Furthermore, data highlight organoids as possible miniatures of organs in term of architectures and distinctive functions. However, it will require additional dedicated efforts to improve potentialities.

Intriguing applications of ccRCC organoid cultures include disease modeling, drug screening and biomarker discovery. Patient-derived models are necessary to improve the knowledge about ccRCC and for the development of new therapeutic approaches. Specific and reliable ccRCC biomarkers for diagnostic, prognostic and therapeutic purposes are needed. The emerging field of personalized oncology requires preclinical tools^78,79^. Many efforts are dedicated to develop meaningful platforms based on the combined use of next-generation DNA-sequencing, ‘Omics’ pipelines and different patient-derived models to devise personalized treatments and identify patients that will most likely benefit from a specific treatment^44,45,80^. Our organoid models may offer new approaches exploiting patient specific molecular alterations in parallel with treatments increasing probability of clinical benefit. Organoid technology may be improved for studying immunotherapy and anti-angiogenesis drugs opening new frontiers of innovations for use of these models^63,64^. Our data showed immune-surveillance marker expression in ccRCC populations and this may foster expectations for development of specific patient-personalized co-cultures for immunotherapy trials.

In the present study we isolated and characterized for the first time organoid cultures from both normal kidney and ccRCC tissues starting from undifferentiated enriched heterogeneous patient cultures. Such organoids may represent very interesting model systems for their use both in vitro and in vivo, with a broad range of potential applications in basic and translational research.

## Supplementary information

Supplementary Information and legends

Supplementary Tables

Supplementary Video 1

Supplementary Video 2

Supplementary Video 3

Supplementary Fig.1

Supplementary Fig.2

Supplementary Fig.3

Supplementary Fig.4

Supplementary Fig.5

Supplementary Fig.6

Supplementary Fig.7

Supplementary Fig.8

Supplementary Fig.9

Supplementary Fig.10

Supplementary Fig.11

Supplementary Fig.12

Supplementary Fig.13

Supplementary Fig.14

Supplementary Fig.15

Supplementary Fig.16

Supplementary Fig.17

Supplementary Fig.18

Supplementary Fig.19

Supplementary File 1

Supplementary File 2

Supplementary File 3

Supplementary File 4
