# Supplementary material for: Organoids as a new model for improving regenerative medicine and cancer personalized therapy in renal diseases
Source: Cell Death Dis. 2019 Feb 27;10(3):201. doi: 10.1038/s41419-019-1453-0 (PMC6393468; doi:10.1038/s41419-019-1453-0)
Supplement: Supplementary file 1 — Supplementary Information and legends [file 41419_2019_1453_MOESM1_ESM.docx]

**SUPPLEMENTARY LEGENDS AND INFORMATION**

**Supplementary Figure Legends:**

**Supplementary Fig.1**

Normal renal freshly and enzymatically dissociated specimens maintained in liquid culture for one week in serum-free stem cell enriching medium^1^ and analyzed for epithelial (EPCAM,CD24), renal specific (CD10), endothelial (CD105), undifferentiated stem cell-like (CD133; CD44) markers by FACS analysis. CD45 staining demonstrated low-level contamination of hematopoietic cells. Background staining was calculated by using appropriate isotype controls. All the cytofluorimetric acquisitions were analyzed by BD FACSDiva Software version 6.1.3 (BD Biosciences).

**Supplementary Fig.2**

(A) Short Tandem Repeat Analysis for sample Identification in organoid and parental derivative tissues and performed with Thermo Fisher-Identifiler kit. Six representative profiles were reported. (B) mRNA expression as assessed by qPCR of Aquaporine 1 (Aqp1), Acquaporin 2 (Aqp2), the ionic channel ClCK1 and Podocin genes in 7 normal kidney organoid cultures.

**Supplementary Fig.3**

(A) Representative patient’s images indicating cortex and medulla area for the specimens selection by microscope. Tumor images was indicated with red line and arrow in both microscope and computed tomography renal images. (B) Heat map representative of mRNA profiles in normal organoid cultures and parental tissues as by RNA-SeQ analysis. (C) Plots reporting mRNA expression id diverse renal areas in normal organoid cultures and parental tissues as by RNA-SeQ analysis. Statistic data analysis was performed with “R” software using non-parametric Wilcoxon test. *P<0.05, **P<0.01, ***P<0.001.

**Supplementary Fig.4**

Plots reporting mRNA expression in diverse renal areas in normal organoid cultures and parental tissues as by RNA-SeQ analysis. Statistic data analysis was performed with “R” software using non-parametric Wilcoxon test. *P<0.05, **P<0.01, ***P<0.001.

**Supplementary Fig.5**

(A) Heat map representative of mRNA profiles in normal organoid cultures when divided for cortex and medulla anatomical derivative areas and analyzed by RNA-SeQ analysis. (B) Cartoon scheme and heat map representative of differential expressed mRNAs evaluated in normal organoid cultures when divided for cortex and medulla anatomical derivative areas and analyzed by RNA-SeQ analysis.

**Supplementary Fig.6**

(A-B) Normal tissue and parental organoid cultures included in cryomatrix OCT, cutted in 8 μM slides and stained with haematoxylin and eosin assay. Representative images were reported. (C) Phase contrast representative of relative organoid cultures analyzed in (B).

**Supplementary Fig.7**

(A) Cartoon scheme summarizing gromerular and tubule area such as discussed in Kidney Organoids: A Translational Journey, Morizane R. Cell. 2017. (B) Fresh fixed and permeabilized organoids evaluated for LTL, WT1, Actin and DAPI markers by immunofluorescence assay. Representative confocal images were reported. (C) Zoom of image in Fig. 2F, representative of organoids were included in cryomatrix (OCT), cutted in 8 μM slides and stained for WT1, TLT, Actin and DAPI markers. Representative confocal images were reported. (D) Fresh fixed and permeabilized organoids evaluated for LTL, WT1, Actin and DAPI markers by immunofluorescence assay. Representative confocal images were reported.

**Supplementary Fig.8**

(A) Plots reporting mRNA expression of gene associated with angiogenesis and evaluated in normal organoid cultures and parental tissues as by RNA-SeQ analysis. Statistic data analysis was performed with “R” software using non-parametric Wilcoxon test. *P<0.05, **P<0.01, ***P<0.001. (B) Fresh fixed and permeabilized organoids evaluated for CD-31, Actin and DAPI markers by immunofluorescence assay. Representative confocal images were reported.

**Supplementary Fig.9**

Representative phase contrast images of normal organoid cultures treated with 20μM and 100μM of Cisplatin 48 hrs after drug exposition. Untreated (NT) cultures were used as control. Two representative samples were reported.

**Supplementary Fig.10**

(A) Western blotting of normal organoid cultures treated with 20μM and 100μM of Cisplatin 48 hrs after drug exposition. Cleaved Caspase-3 activation and CD-31 protein were evaluated. GAPDH protein was used as internal control. Untreated (NT) cultures were used as control. Two representative samples were reported. (B) CD-31 antigen localization in normal organoid cultures treated with 100μM of Cisplatin 48 hrs after drug exposition and evaluated by immunofluorescence assay. Untreated (NT) cultures were used as control. Representative images were reported.

**Supplementary Fig.11**

Cleaved-Caspase 3, Actin, and DAPI markers evaluated by immunofluorescence analysis and in combination with Tunel assay performed in normal organoid cultures treated with 100μM of Cisplatin at 72 hrs of drug exposition. Untreated (NT) cultures were used as control. Representative images were reported. White arrows indicate areas of cell death.

**Supplementary Fig.12**

Cleaved-Caspase 3, Actin, WT1 and DAPI markers evaluated by immunofluorescence assay in normal organoid cultures treated with 100μM of Cisplatin at 72 hrs of drug exposition. Untreated (NT) cultures were used as control. Representative images were reported.

**Supplementary Fig.13**

(A) DAPI staining for nuclei evaluation of renal cancer organoids. Scale bars 100μm. (B) Reverse phase protein array (RPPA) analysis of normal and cancer renal populations one week after enzymatic and mechanic dissociation culture in serum-free stem cell enriching medium. Graphs and table report several significant and differentially expressed antigens when normal and tumor cultures were compared. Non-parametric Wilcoxon test (JMP11) was performed.

**Supplementary Fig.14**

(A-B) Gene allele frequency (FeQ) and variant multi-clone proportion evaluated by WES genome sequencing in tumor organoids versus tumor parental tissues. (A) Venny diagram of one sample of three was reported (Tissue: tumor tissue; Organoid: Tumor organoid). Homozygous­_Ref: homozygote wild type allele; Heterozygous: heterozygote allele variant; Homozygous­_Alt: homozygote allele variant (B) Two of three tumor organoid and tissue coupled analysis were reported. 0/0: homozygote wild type allele; 0/1: heterozygote allele variant, 1/1: homozygote allele variant. (C-D) Paired normal and tumor tissues analyzed and compared to their organoid cultures counterparts for most renal cancer frequent mutations. (C) On the left, cartoon representing mutational analysis scheme. (D) On the Right, tables reported mutated genes raised and shared by WES genome analyses.

**Supplementary Fig.15**

(A)Tumor organoid cultures and their normal counterpart were exposed to different doses of Sutent and Tensirolimus for 72 hrs. Drug efficacy driver genes, pERK T202/Y204, mTOR S2448, VEGFR2 Y996, were evaluated by Western blotting. Cyclin D1 protein was evaluated for proliferation analysis by Western blotting. GAPDH protein was used as internal control. (B) Phase contrast images of tumor organoid cultures were exposed to different doses of Sutent and Tensirolimus for 72 hrs. Untreated (NT) normal and tumor organoid cultures were shown. Untreated (NT) normal counterpart only was reported, in fact, different doses of Sutent and Tensirolimus after 72 hrs did not affected normal cultures (Data not shown). Graph reports number of RCC organoids after drug treatment and reported as percentage over relative NT.

**Supplementary Fig.16**

Representative phase contrast images of normal organoid cultures treated with SU11274 10 µM, FORETINIB 10 μM, LENVATINIB+EVEROLIMUS 10 µM, Cabozantinib 10 μM.

and analyzed 72 hrs after drug exposition. Untreated (NT) cultures were used as control. Parental normal counterpart cultures of RCC cultures reported in Supplementary Fig.17 were shown.

**Supplementary Fig.17**

Representative phase contrast images of RCC organoid cultures treated with SU11274 10 µM, FORETINIB 10 μM, LENVATINIB+EVEROLIMUS 10 µM, Cabozantinib10 μM and analyzed 72 hrs after drug exposition. Untreated (NT) cultures were used as control.

**Supplementary Fig.18**

(A) Analysis of most frequent mutations in renal cancer evaluated in two specific cases. Tumor organoid cultures were compared with parental tumor tissues by PCR and Sanger sequencing system. (See Supplementary information for mutation details). (B) Representative Western blotting images of untreated (NT) and treated RCC and parental normal organoid cultures with SU11274 10 µM, FORETINIB 10 nM, LENVATINIB+EVEROLIMUS 10 µM, Cabozantinib10 nM, analyzed 72 hrs after drug exposition by Western blotting. Drug efficacy driver genes, , pERK T202/Y204, pAKT S473 were evaluated. Cleaved Caspase-3 protein was evaluated as apoptosis induction. GAPDH were used as internal control. (C) Representative Western blotting images of an additional untreated (NT, line 1) and treated RCC organoid cultures with SU11274 10 μM, FORETINIB 10 nM, LENVATINIB+EVEROLIMUS 10 μM, Cabozantinib10 nM, analyzed 72 hrs after drug exposition. Drug efficacy driver genes, pERK T202/Y204, pAKTS473 were evaluated. Cleaved-Caspase 3 protein was evaluated as apoptosis induction. GAPDH were used as internal control. Parental normal counterpart (NT, line 6) was reported as control.

**Supplementary Fig.19**

Reverse phase protein array stainings performed on previously published cohort (di Martino et al. 2018). PD-L1 and PD-L2 staining were run and a new analysis was performed including previously reported IL-6 and RANKL (di Martino et al. 2018). Blue line represents arbitrary high (Pink triangles) and low (Black circles) PD-L1 levels. Black circles and Pink triangles in the RANKL, PD-L2 and IL-6 antigen boxes indicates RCC cases separated for PDL-1 level highlighting co-expressions or relative exclusions.

**Supplementary movie legends**

**Supplementary movie 1-2:** Immunofluorescence staining for actin (red) of a normal organoid culture analyzed by Confocal z-stack. Nuclei are stained with DAPI (blue). Microscope magnification 20x. Scale bars 100μm. Z-dimensions: 33 slices, 1,160um/per slice.

**Supplementary movie 3:** Confocal z-stack of a ccRCC G4 organoid culture stained by immunofluorescence for actin (red). Nuclei are stained with DAPI (blue). Microscope magnification 20x. Scale bars 100μm. Z-dimensions: 11 slices, 1,160μm/per slide.

**Supplementary table Legends**

**Supplementary Table1**

Table reports organoid specific medium components.

**Supplementary Table2**

Table summarizing the clinical features of the patients whose tissues were used to derive cancer and their normal control part for organoid culture establishment. G refers to the Grading. The stage is assigned following the TMN staging system for Kidney tumors. n/a: not applicable; N/A: not available. (WHO 2016).

**Supplementary Table3:**

Short Tandem Repeat Analysis for sample Identification in tumor organoid and parental derivative tissues and performed with Thermo Fisher-Identifier kit. Three representative profiles of tumor organoid and parental tissues were reported (Upper panel). Representative profiles of two paired tumor and normal organoid cultures and relative parental tissues were reported (Down panel).

**Supplementary Table 4:**

Wild Type gene allele frequency (FeQ) and variant multi-clone proportion evaluated by WES genome sequencing in normal organoids versus normal parental tissue analysis of three normal organoid and tissues coupled samples were reported. 0/0: homozygote wild type allele; 0/1: heterozygote allele variant, 1/1: homozygote allele variant.

**Supplementary Table5:**

Table summarizing the clinical features of the patient whose frozen tissues were used to derive cancer organoid cultures. G refers to the ISUP 2013 and WHO 2016 guidelines. The stage is assigned following the TMN staging system for Kidney. n/a: not applicable; N/A: not available.

**Supplementary Table 6:**

Table summarizing the clinical features of the cancer organoid cultures implanted in immunocompromized mice. G refers to the ISUP 2013 and WHO 2016 guidelines. The stage is assigned following the TMN staging system for Kidney. n/a: not applicable; N/A: not available.

**Supplementary information**

**Sanger sequencing**

Primer pairs and annealing temperatures used to amplify the selected *PBMR1*, *TSC2*, *VHL*, *KDM5C*, *SETD2* and *PIK3C2A* coding regions, and sizes of PCR products.

| **Gene** | **Variants position** | **Primer Sequence (5′→3′)** | | **T_ann_.(°C)** | **bp** |
| --- | --- | --- | --- | --- | --- |
|  | | **Forward** | **Reverse** |  |  |
| *PBMR1* | 52696147  52696217 | GCACAGGAATTCTAGTTGCC | GTCTTTGCTGAAACAGGTGC | 60 | 338 |
| *TSC2* | 2130198 | GCTGGTGGTTTTGCATCAGG | CCAGCCCTGGGTCAGCAG | 63 | 258 |
| *VHL* | 10191623  10191611  10191528 | CCAGTGTATACTCTGAAAGAG | CTCTGAGAATGAGACACTTTG | 57 | 297 |
| *VHL* | 10183862  10191528 | GAGGAGGAGATGGAGGCC | GGCTTCAGACCGTGCTATC | 60 | 246 |
| *KDM5C* | 53240810 | CCGTTTACTGTCACTGACAG | GGTATTGATGCCAGATGGTC | 58 | 227 |
| *SETD2* | 47125518 | CAGATGTAGGTAGCTTACTAG | TCCACCAAGCTGAGCACAG | 60 | 277 |
| *PIK3C2A* | 17190783 | CTGGACGATAGATAGGTAAGC | GAGGACAGTGGCCACCTGG | 63 | 256 |

Follows Sequence chromatograms showing the occurrence of somatically acquired VHL mutation in cancer tissue and organoid cells. A reference sequence is reported.


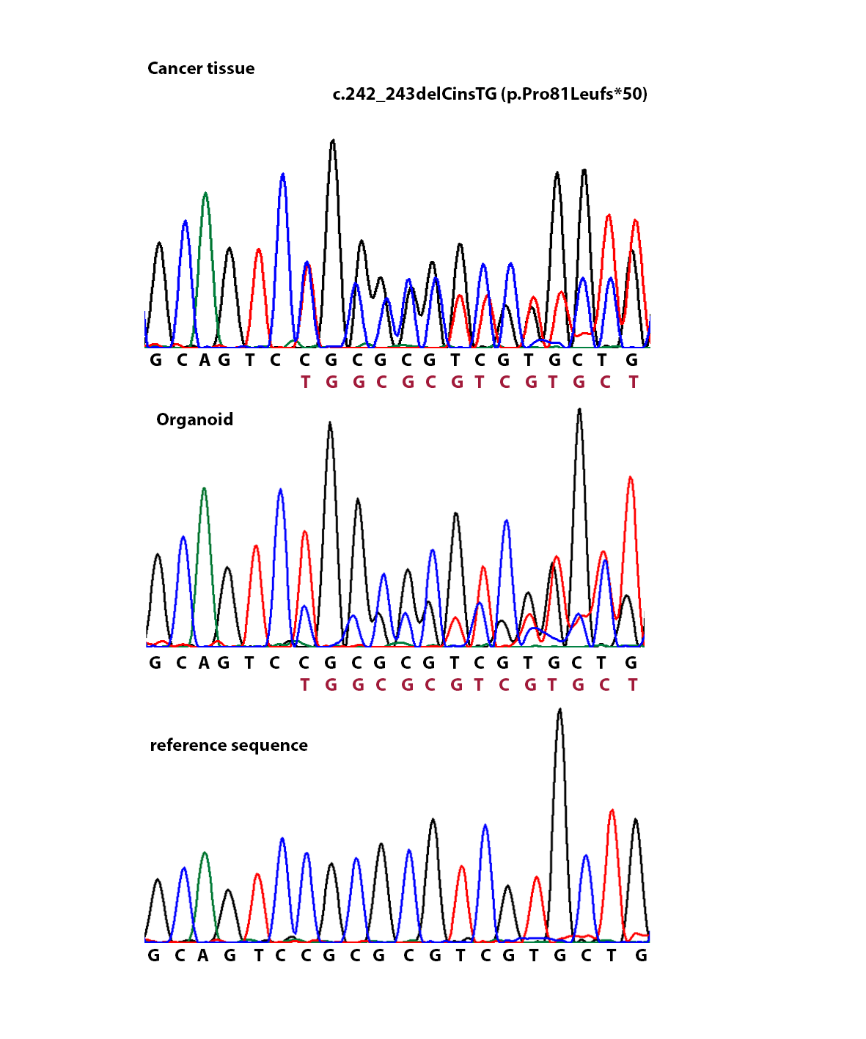


**Supplementary Materials and Methods**

***Immunofluorescence, cytochemistry and Western Blotting***

For immunofluorescence, organoids were fixed in 2% paraformaldehyde and permeabilized in 0.1% Triton X-100 (Bio-Rad) then incubated overnight at 4°C with primary antibodies dissolved in PBS containing 3% BSA (Bio-ad), 0.1% Triton X-100. The following antibodies were used: goat anti-human SOX2 (R&D Systems; AF2018), mouse anti-human Ck8-18 (Abcam; ab17139), rabbit anti-human E-cadherin (Cell Signaling; 3195), mouse anti-human Ki67 (DAKO; M7240), rabbit anti-human Aquaporin 1 (Abcam; ab15080), rabbit anti-human Aquaporin 2 (GeneTex; GTX31904) and mouse anti-human HIF1α (Abcam; ab16066), rabbit anti-WT1 ( sc-192, Santa Cruz Biotechnology), LTL-biotin-conjugated ( B-1325, Vector Laboratories), rabbit anti-Cleaved-Caspase 3 (9661S, Cell Signaling Technology) tunel assay in situ cell death detection kit, TMR red (12156792910 Roche). After two washes in DPBS, cells were incubated with Alexa Fluor-conjugated secondary antibodies and Alexa Fluor 555 phalloidin (Thermo Fisher; A34055) for 30 minutes at room temperature in the dark, stained for 15 minutes with 4,6-diamidino-2-phenylindole (DAPI) (Invitrogen), diluted in DPBS 3% BSA, and mounted with Prolong-Gold antifade (Invitrogen). Slides were analyzed on a FV1000 confocal microscope (Olympus). Haematoxylin and Eosin Cytochemical analyses were performed on murine frozen tissues and organoids. For the latter, the cultures were harvested, pelleted, resuspended in Matrigel (Corning), OCT (Tissue-Tek) and cryopreserved. Tissue slices of 2 μm thickness, were fixed in 2% paraformaldehyde, washed with DPBS and dH_2_O. Murine tissue staining was performed by incubating for 30 seconds with Haematoxylin (Sigma-Aldrich), followed by wash with tap water for 15 seconds, a rinse in dH_2_O and ethanol 95% prior to 30 seconds incubation with Eosin (Sigma-Aldrich) and a final wash with DPBS. Slides were air-dried and mounted with DPX (Sigma-Aldrich). Organoids cultures and parental tissues were embedded in cryomatrix OCT and cutted in 8 μM slides with cryostat sectioning ( Thermo Scientific). For hematoxylin and eosin staining we used a standard protocol^1^ for both tissues and organoids. Cryomatrix OCT embedded organoid were cutted in 8 μM slides with cryostat sectioning ( Thermo Scientific) and stained as above by immunofluorescence.

For Western Blotting, cellular pellets were lysed in Tissue Protein Extraction (T-PER) buffer (Thermo Scientific, Rockford, IL), 300 mM NaCl, protease and phosphatase inhibitors (Sigma-Aldrich Inc., Saint Louis, MO). Cell lysates were subjected to gel electrophoresis using a 4-12% NuPAGE SDS-PAGE Gel System (Invitrogen, Pasley, UK) and transferred to nitrocellulose membrane (Thermo Scientific, Rockford, IL). Membrane was blocked for 45 min with 5% non-fat dry milk (Bio-RAD) in TBS containing 0.2% Tween-20 and incubated over night with primary antibody (1:1000) diluited in BSA 5%. The primary antibodies used are: CD31 (Dako MO823), Cyclin D1 (BD 554180), Cleaved-Caspase 3 (9662), pAKT S473 (4058), pERK T202/Y204 (9101), VEGFR2 Y996 (2474), mTOR S2448 (5536) (all from Cell Signaling Technology, Danvers, MA) and GAPDH (G9545) (Sigma-Aldrich Inc, Saint Louis, MO). Washed filters were then incubated for 45 min with HRP-conjugated anti-rabbit or anti-mouse secondary antibodies (Amersham Pharmacia Biotech, Piscataway, NJ) and visualized by using an enhanced chemioluminescence detection system (ECL system (Thermo Scientific), G:Box (Syngene) with Image Lab software).

***RNA extraction and Real-time PCR analysis***

RNA was extracted using Trizol reagent (Thermo Fisher) following the manufacturers' protocol. An amount of RNA corresponding to 1 µg was reverse-transcribed with M-MLV reverse transcriptase (Invitrogen) with random primers. cDNA was diluted 1:10 in the PCR reactions. The gene expressions levels were measured using the Taqman expression assay for PGK1, NDRG1, VEGFR2, CAIX, Aquaporin 1, Aquaporin 2, ClC-K1, THP, Podocin and GAPDH (Invitrogen) following the manufactures' instruction. All the experiments were performed on a StepOne instrument (Applied Biosystems) and results were expressed as 2^-ΔΔCT^, where ΔΔCT = ΔCT sample - ΔCT calibrator and ΔCT is the difference in threshold cycles (CT) between the mRNA and GAPDH amplicons. Real-time PCR analyses were performed by using the ABI PRISM 7700 Sequence Detector software (Applied Biosystem) whereby the CT values are automatically calculated via negative correlation with internal assay reference.

***Reverse-phase Protein Arrays***

*Reverse-Phase Protein Arrays (RPPA) were conducted as previously described (1). Additional immunostaining was carried out using a signal amplification kit (DAKO, Carpinteria, CA). Overall, previously printed slides were probed with primary antibody against PD-L1 (13684) and PD-L2 (82723) (both from Cell Signaling Technology, Danvers, MA). Primary antibody binding was detected using a biotinylated goat anti-rabbit IgG H+L (1:7500) (Vector Laboratories, Burlingame, CA) followed by streptavidin-conjugated IRDye680 fluorophore (LI-COR Biosciences, Lincoln, NE). Primary antibodies were previously validated for single band specificity by Western Blot using cell lysates. Negative control slides were incubated with secondary antibody alone. All immunostained slides were scanned using a Tecan power scanner™ (Tecan Group Ltd, Switzerland). Acquired images were analyzed with MicroVigene v5.0. (VigeneTech, Carlisle, MA) for spot detection, local background subtraction, negative control subtraction, replicate averaging and total protein normalization.*

***In vivo procedures: injection under the renal capsule***

Six weeks old (weight≈20gr) NOD Cg-Prkdc^scid^ Il2rg^tm1Wjl^/SzJ (NSG) mice (Charles River Laboratories) were used for the injections. Depending on the gender of the original patient, male or female mice were used. The farming was carried out in ventilated cages hosting groups of four individuals with food and water provided ad libitum. All animal procedures were performed according to the protocol approved by the Animal Care Committee of Italian National Institute of Health. For injections under the kidney capsule, animals were anesthetized with a mixture of ketamine (100 mg/kg, Intervet Productions) and xylazine (10 mg/kg, Bayer). Under sterile conditions, a skin incision of approximately 1 cm was made along the dorsal midline. With the mouse lying on its side, a body wall incision was then cut slightly shorter than the long axis of the kidney. The left kidney was slipped out of the body by applying pressure on both sides of the organ. Injection of pooled organoids (approximately composed of 1×10^5^ cells) diluted in 20 µl of Matrigel (Corning) was performed with a 29-G needle in the sub-capsular space of the kidney. The kidney was then gently eased back into the peritoneal space; the body wall incision was closed using a 4/0 absorbable suture (Ethicon), while the skin incision was closed with surgical staples (Fine Science Tools). A volume of 1 ml of saline solution was administered subcutaneously immediately after surgery.

***Cell lines***

The human renal cell cancer 786-0 were obtained from the ATCC, criopreserved at low passages and never kept in culture for more than 6 months. Cells were cultured as monolayers at 37 °C and 5% CO2 in RPMI-1640 (Thermo Fisher), GlutaMAX (Gibco-Invitrogen) supplemented with 10% fetal bovine serum (FBS) (Gibco- Invitrogen).

**Whole exome sequencing**

Targeted enrichment and massively parallel sequencing experiments were performed on genomic DNA extracted from tissues and organoid cultures, by means of Illumina Nextera Rapid Hiseq 2500 [DNA Link Sequencing service (www.dnalinkseqlab.com)] sequencer. Exome capture was carried out using Twist Human Core Exome Kit (Twist Bioscience). The data analysis was performed using an in-house implemented pipeline, which mainly takes advantage of the Genome Analysis Toolkit (GATK V.3.7) ^2^framework, as previously reported ^3,4^. In detail, reads mapping was performed by BWA V.0.7.11 ^5^, and GATK tools were used for base quality recalibration and variants calling. Afterwards, germinal and somatic SNVs/INDELs were identified by means of the GATK’s Haplotype Caller and MuTect2 tools, respectively; finally, variants will be quality-filtered, according to GATK’s 2016 best practices. SnpEff toolbox (V.4.3) ^6^ was used to predict the functional impact of variants, which were filtered to retain only those located in exons with any effect on the coding sequence, and splice site regions (variants located from −3 to +8 with respect to an exon-intron junction). Moreover, functional annotation of variants was performed using SnpEff and dbNSFP (V.3.5) ^6-8^. WES statistics are reported in Supplementary Data.

**Bibliography**

1 di Martino, S. *et al.* Renal cancer: new models and approach for personalizing therapy. *J Exp Clin Cancer Res* **37**, 217, doi:10.1186/s13046-018-0874-4 (2018).

2 McKenna, A. *et al.* The Genome Analysis Toolkit: a MapReduce framework for analyzing next-generation DNA sequencing data. *Genome Res* **20**, 1297-1303, doi:10.1101/gr.107524.110 (2010).

3 Kortum, F. *et al.* Mutations in KCNH1 and ATP6V1B2 cause Zimmermann-Laband syndrome. *Nat Genet* **47**, 661-667, doi:10.1038/ng.3282 (2015).

4 Sferra, A. *et al.* TBCE Mutations Cause Early-Onset Progressive Encephalopathy with Distal Spinal Muscular Atrophy. *Am J Hum Genet* **99**, 974-983, doi:10.1016/j.ajhg.2016.08.006 (2016).

5 Li, H. & Durbin, R. Fast and accurate long-read alignment with Burrows-Wheeler transform. *Bioinformatics* **26**, 589-595, doi:10.1093/bioinformatics/btp698 (2010).

6 Cingolani, P. *et al.* A program for annotating and predicting the effects of single nucleotide polymorphisms, SnpEff: SNPs in the genome of Drosophila melanogaster strain w1118; iso-2; iso-3. *Fly (Austin)* **6**, 80-92, doi:10.4161/fly.19695 (2012).

7 Dong, C. *et al.* Comparison and integration of deleteriousness prediction methods for nonsynonymous SNVs in whole exome sequencing studies. *Hum Mol Genet* **24**, 2125-2137, doi:10.1093/hmg/ddu733 (2015).

8 Liu, X., Jian, X. & Boerwinkle, E. dbNSFP v2.0: a database of human non-synonymous SNVs and their functional predictions and annotations. *Hum Mutat* **34**, E2393-2402, doi:10.1002/humu.22376 (2013).
