# Supplementary material for: Organoids as a new model for improving regenerative medicine and cancer personalized therapy in renal diseases
Source: Cell Death Dis. 2019 Feb 27;10(3):201. doi: 10.1038/s41419-019-1453-0 (PMC6393468; doi:10.1038/s41419-019-1453-0)
Supplement: Supplementary file 2 — Supplementary Tables [file 41419_2019_1453_MOESM2_ESM.pdf]

Supplementary Table 1

**ADMEM F12**	Invitrogen
**GFR Matrigel**	Corning
**GlutaMax**	Invitrogen
**N- acetyl-L-cysteine**	Sigma-Aldrich
**Primocin**	invivoGen
**B27 Supplement**	Invitrogen
**Pennicillin/streptomycin**	Lonza
**Human recombinant FGF 2**	Peprotech
**Human recombinant EGF**	Peprotech
**HEPES Buffer**	Sigma
**Rho kinase inhibitor Y-27632**	Sellenckchem
**A83-01 inibitor TGFbeta**	Tocris

Supplementary Table 2: Clinical features of the patients enrolled

Sample	Sex	Diagnose	ISUP Grade	pT	pN	M	Stage
1	f	ccRCC	G2	T1b	Nx	M0	1
2	f	ccRCC	G3	T3a	Nx	M1	4
3	m	ccRCC	G2	T3a	N0	M1	4
4	m	ccRCC	G3	T3b	N2	M1	4
5	m	ccRCC	G3	T2a	Nx	M1	4
6	m	ccRCC	G4	T3a	Nx	M0	3
7	m	ccRCC	G4	T1b	Nx	M1	4
8	f	ccRCC	G4	T3a	Nx	M1	4
9	f	ccRCC	G3	T3a	Nx	M0	3
10	m	ccRCC	G2	T1b	Nx	M0	1
11	m	ccRCC	G3	T3a	Nx	M0	3
12	m	ccRCC	G3	T2a	Nx	M0	2
13	f	ccRCC	G3	T2a	Nx	M0	2
14	f	ccRCC	G2	T1a	Nx	M0	1
15	m	ccRCC	G2	T1b	Nx	M0	1

Supplementary Table 3:Short Tandem Repeat Analysis

Type:Tumor Samples	D8S1179	D21S11	D7S820	CSF1PO	D3S1358	Th01	D13S317	D16S539	D2S1338	D19S433	vWA	TPOX	D18S51	Amel	D5S818	FGA
Tissue 1	15;15	29;31.2	11;12	10;10	17;18	6;9.3	10;11	12;13	18;20	13;15	17;17	8;8	17;17	XY	11;12	20;21
Organoid Culture 1	15;15	29;31.2	11;12	10;10	17;18	6;9.3	10;11	12;13	18;20	13;15	17;17	8;8	17;17	XY	11;12	20;21
Tissue 2	15;16	29;31	8;10	10;12	15;16	29;31	8;10	10;12	17;25	12;15	15;17	8;11	12;17	XX	9;11	21;25
Organoid Culture 2	15;16	29;31	8;10	10;12	15;16	7;9	8;8	12;12	17;25	12;15	15;17	8;11	12;17	XX	9;11	21;25
Tissue 3	9;13	30;30	8;8	12;12	16;16	7;7	10;10	11;13	23;23	13;13	15;17	8;8	14;14	XX	11;11	21;25
Organoid Culture 3	9;13	30;30	8;8	12;12	16;16	7;7	10;10	11;13	23;23	13;13	15;17	8;8	14;14	XX	11;11	21;25

Type: Normal and Tumor Samples	D8S1179	D21S11	D7S820	CSF1PO	D3S1358	Th01	D13S317	D16S539	D2S1338	D19S433	vWA	TPOX	D18S51	Amel	D5S818	FGA
Tumor Tissue 4	12;13	30;31.2;32.2	8;11	11;12	15;18	9;10	9;12	11;13	18;23	14;14	17;18	8;10	12;14	XX	10;11	20;25
Tumor Organoid Culture 4	12;13	30;31.2;32.2	8;11	11;12	15;18	9;10	9;12	11;13	18;23	14;14	17;18	8;10	12;14	XX	10;11	20;25
Normal Tissue 4	12;13	30;32.2	8;11	11;12	15;18	9;10	9;12	11;13	18;23	14;14	17;18	8;10	12;14	XX	10;11	20;25
Normal Organoid Culture 4	12;13	30;32.2	8;11	11;12	15;18	9;10	9;12	11;13	18;23	14;14	17;18	8;10	12;14	XX	10;11	20;25
Tumor Tissue 5	14;15	29;32.2	8;9	11;12	15;17	6;8	9;11	11;11	18;18	15;16.2	17;17	8;9	12;13	XY	12;13	23;26
Tumor Organoid Culture 5	14;15	29;32.2	8;9	11;12	15;17	6;8	9;11	11;11	18;18	15;16.2	17;17	8;9	12;13	XY	12;13	23;26
Normal Tissue 6	14;15	29;32.2	8;9	11;12	15;17	6;8	9;11	11;11	18;18	15;16.2	17;17	8;9	12;13	XY	12;13	23;26
Normal Organoid Culture 6	14;15	29;32.2	8;9	11;12	15;17	6;8	9;11	11;11	18;18	15;16.2	17;17	8;9	12;13	XY	12;13	23;26

Supplementary Table 4: Gene Allele frequency and multiclonal variant representation

Allele FeQ	Shared with Normal Tissue (Number of analyzed genes)	Only normal Organoid (Number of analyzed genes)
0/0	195	297
0/1	9849	288
1/1	5294	126

Supplementary Table 5: Clinical features frozen samples

Sample	Sex	Diagnose	Grade	pT	pN	M	Stage
1	f	ccRCC	G2	T3a	Nx	M0	3
2	m	ccRCC	G3	T3a	N0	M0	3
3	m	ccRCC	G4	T1b	Nx	M0	1
4	f	ccRCC	G3	T3a	N0	M0	3
5	m	ccRCC	G4	T4	N0	M0	4
6	m	ccRCC	G4	T3a	N2	M1	4
7	m	ccRCC	G4 left/G3 right	T3a left/T1a right	N0	M1	4
8	m	ccRCC	G4	T3a	N0	M0	3

Supplementary Table 6: Cancer organoid implanted in immunocompromized mice

Sample	Sex	Diagnose	Grade	pT	pN	M	Stage	Kidney	Recurrence	Tumorgraft
1	f	ccRCC	G3	T3a	Nx	M1	4	right	YES	YES
2	m	ccRCC	G4	T3a	Nx	M0	3	left	YES	YES
3	m	ccRCC	G4	T3a	N2	M1	4	right	N/A	YES
4	m	ccRCC	G4	T3a	N0	M0	3	left	NO	NO
