# Supplementary figures and images for: Organoids as a new model for improving regenerative medicine and cancer personalized therapy in renal diseases
Source: Cell Death Dis. 2019 Feb 27;10(3):201. doi: 10.1038/s41419-019-1453-0 (PMC6393468; doi:10.1038/s41419-019-1453-0)

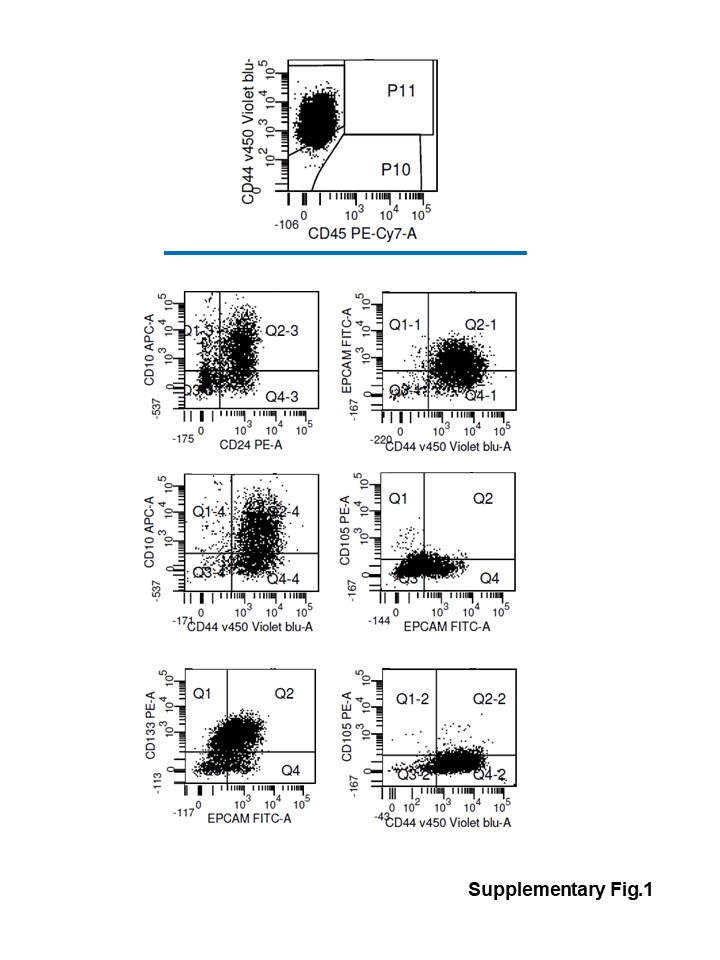

Supplement: Supplementary file 6 — Supplementary Fig.1 [file 41419_2019_1453_MOESM6_ESM.jpg]

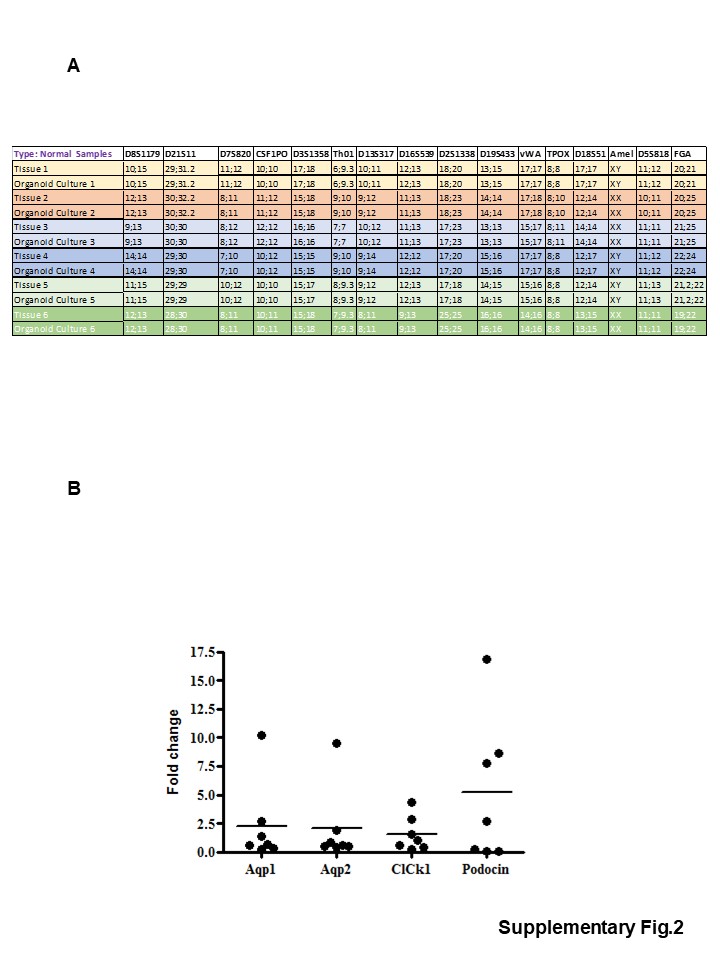

Supplement: Supplementary file 7 — Supplementary Fig.2 [file 41419_2019_1453_MOESM7_ESM.jpg]

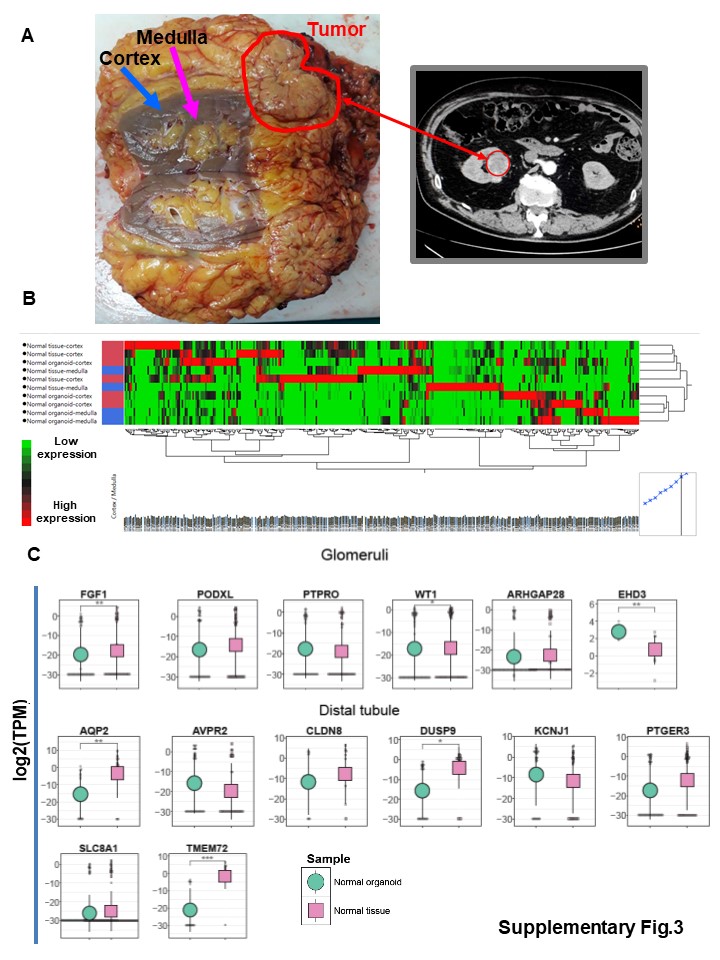

Supplement: Supplementary file 8 — Supplementary Fig.3 [file 41419_2019_1453_MOESM8_ESM.jpg]

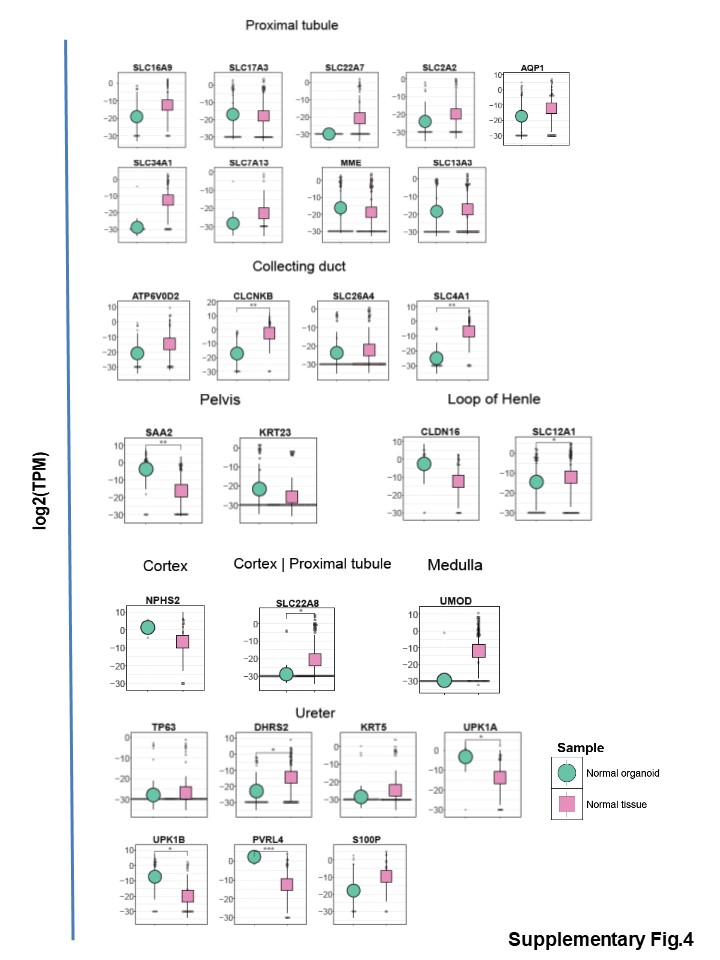

Supplement: Supplementary file 9 — Supplementary Fig.4 [file 41419_2019_1453_MOESM9_ESM.jpg]

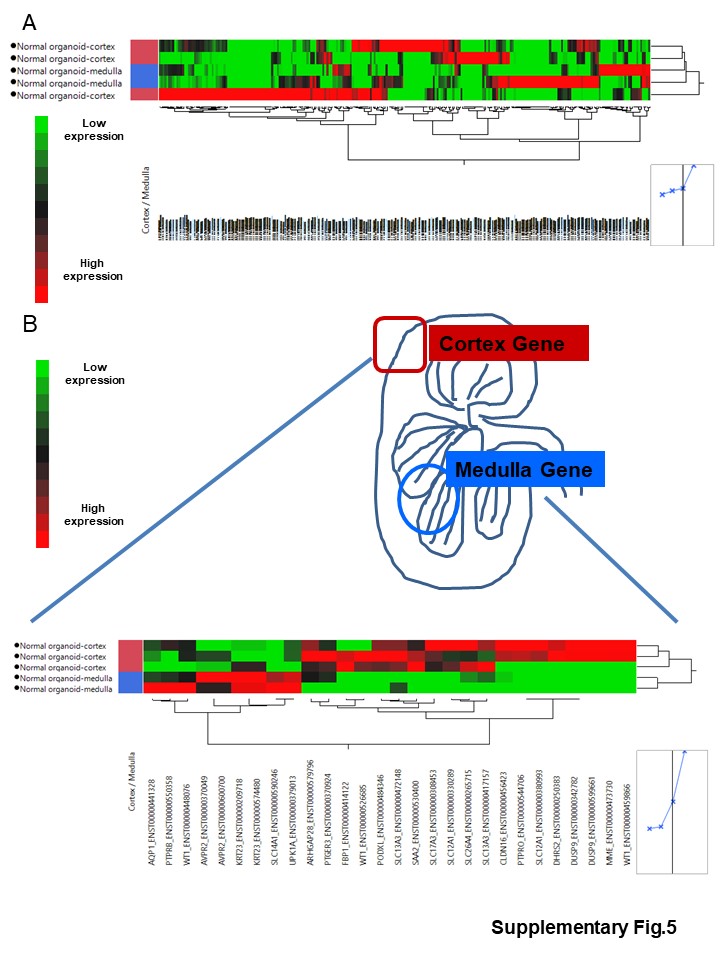

Supplement: Supplementary file 10 — Supplementary Fig.5 [file 41419_2019_1453_MOESM10_ESM.jpg]

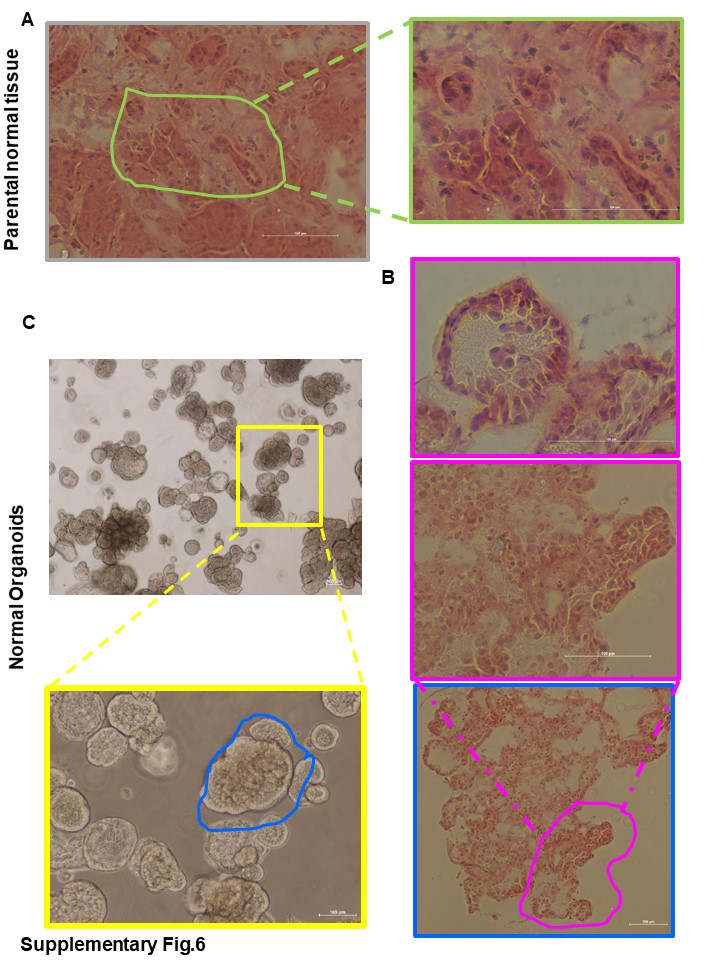

Supplement: Supplementary file 11 — Supplementary Fig.6 [file 41419_2019_1453_MOESM11_ESM.jpg]

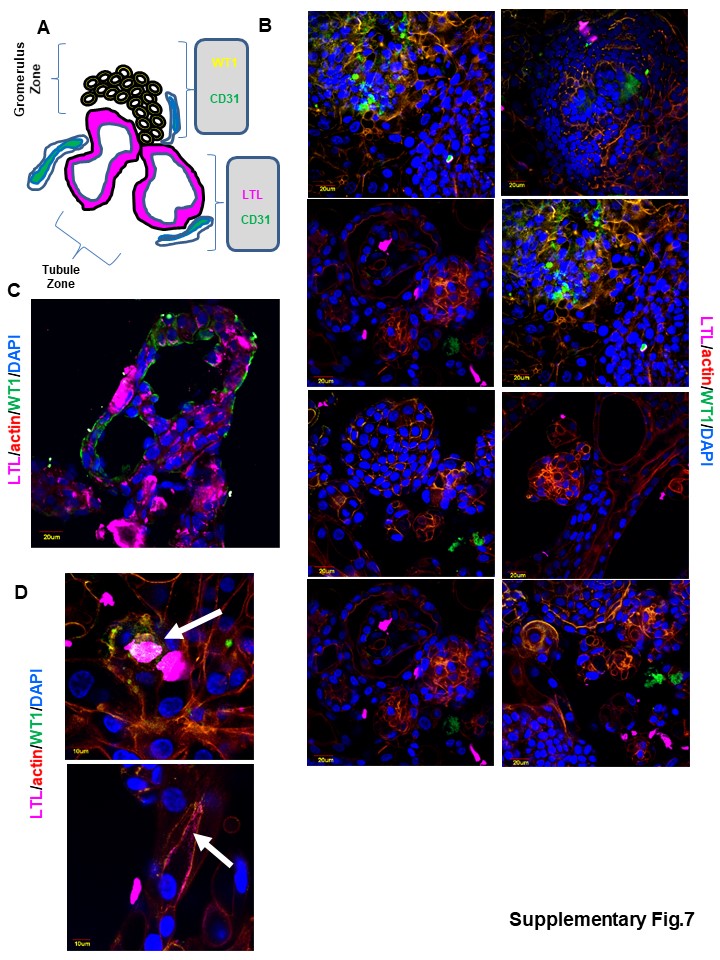

Supplement: Supplementary file 12 — Supplementary Fig.7 [file 41419_2019_1453_MOESM12_ESM.jpg]

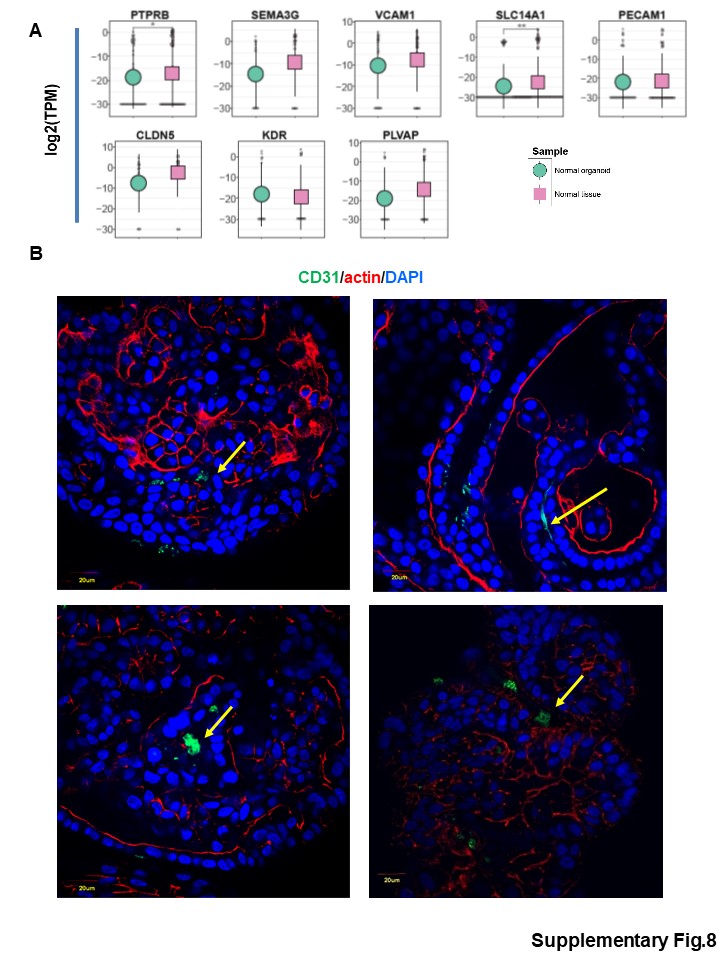

Supplement: Supplementary file 13 — Supplementary Fig.8 [file 41419_2019_1453_MOESM13_ESM.jpg]

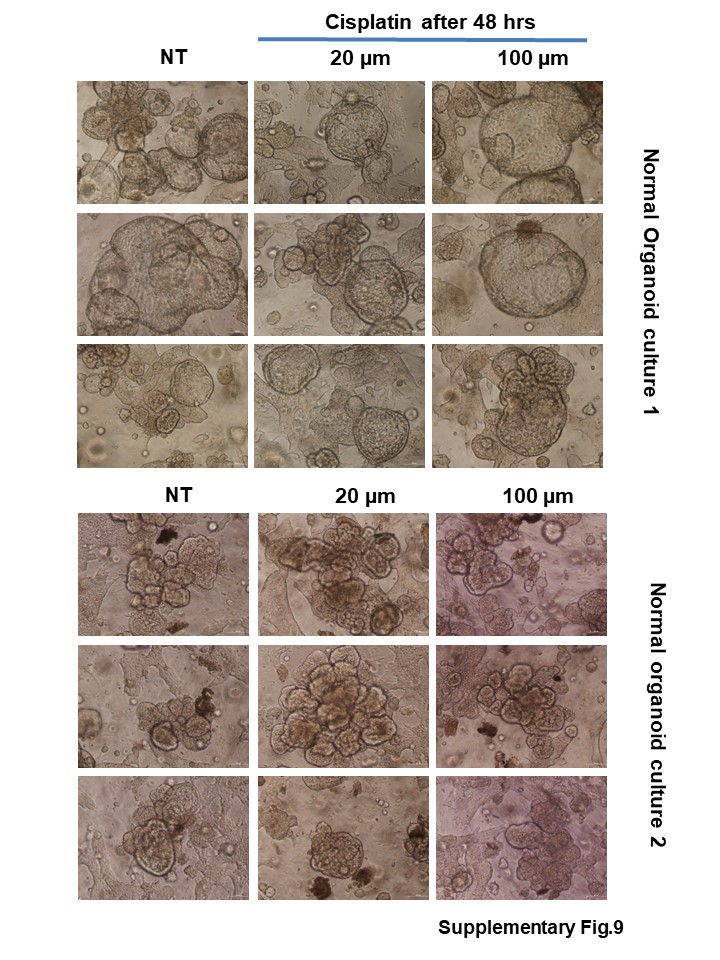

Supplement: Supplementary file 14 — Supplementary Fig.9 [file 41419_2019_1453_MOESM14_ESM.jpg]

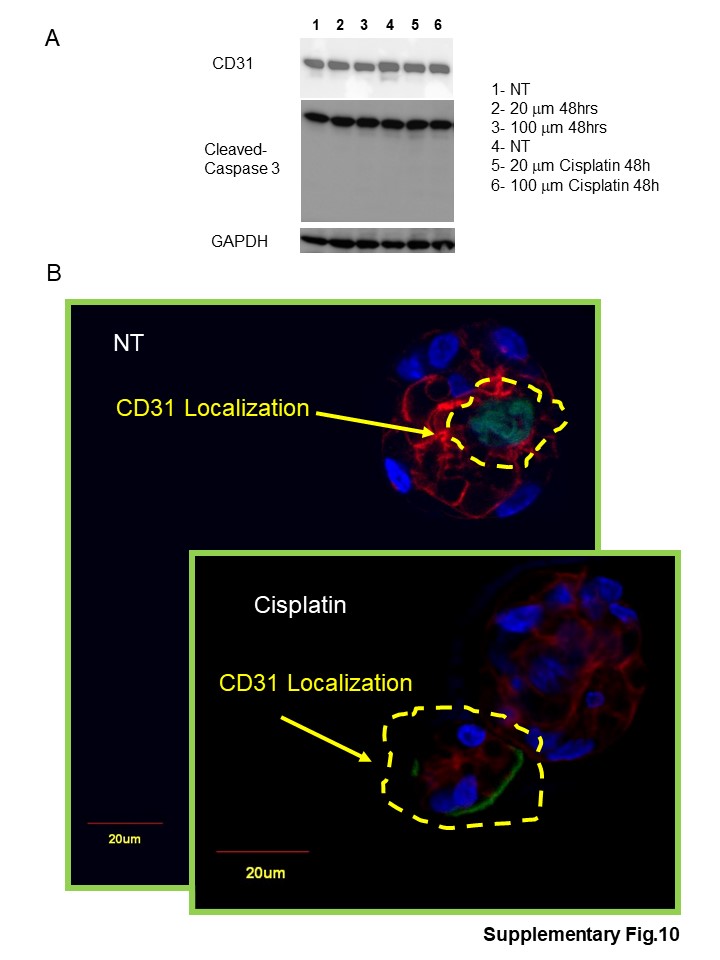

Supplement: Supplementary file 15 — Supplementary Fig.10 [file 41419_2019_1453_MOESM15_ESM.jpg]

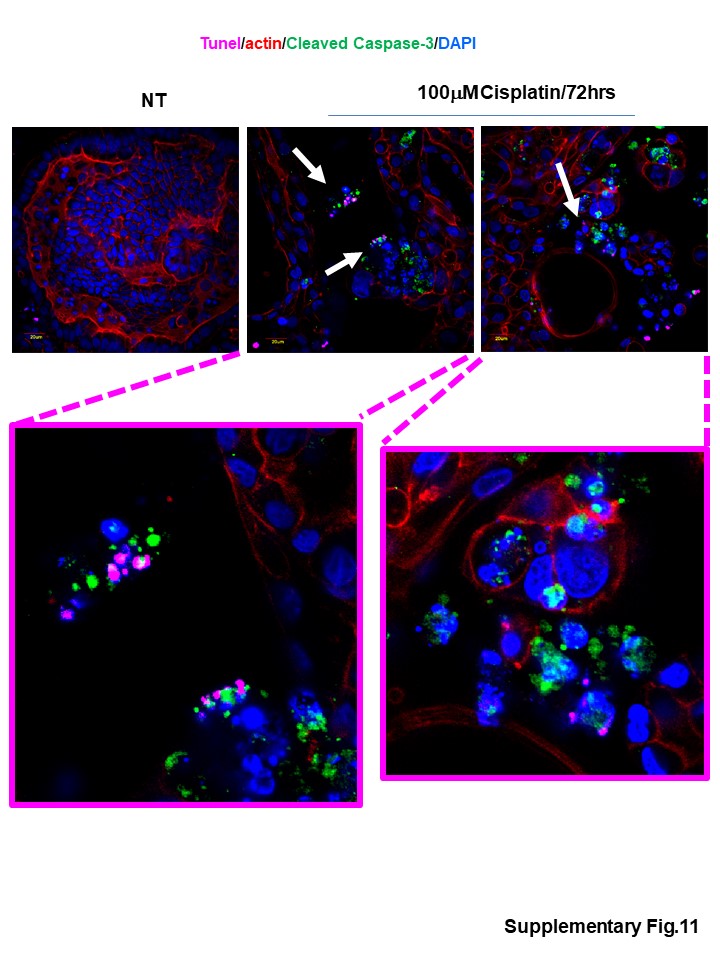

Supplement: Supplementary file 16 — Supplementary Fig.11 [file 41419_2019_1453_MOESM16_ESM.jpg]

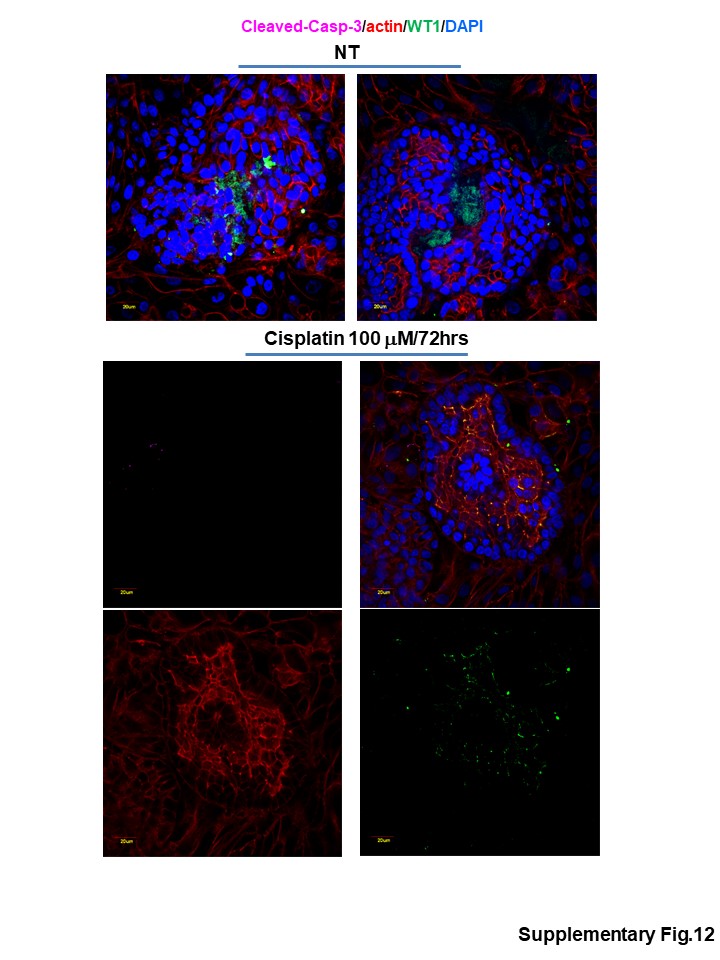

Supplement: Supplementary file 17 — Supplementary Fig.12 [file 41419_2019_1453_MOESM17_ESM.jpg]

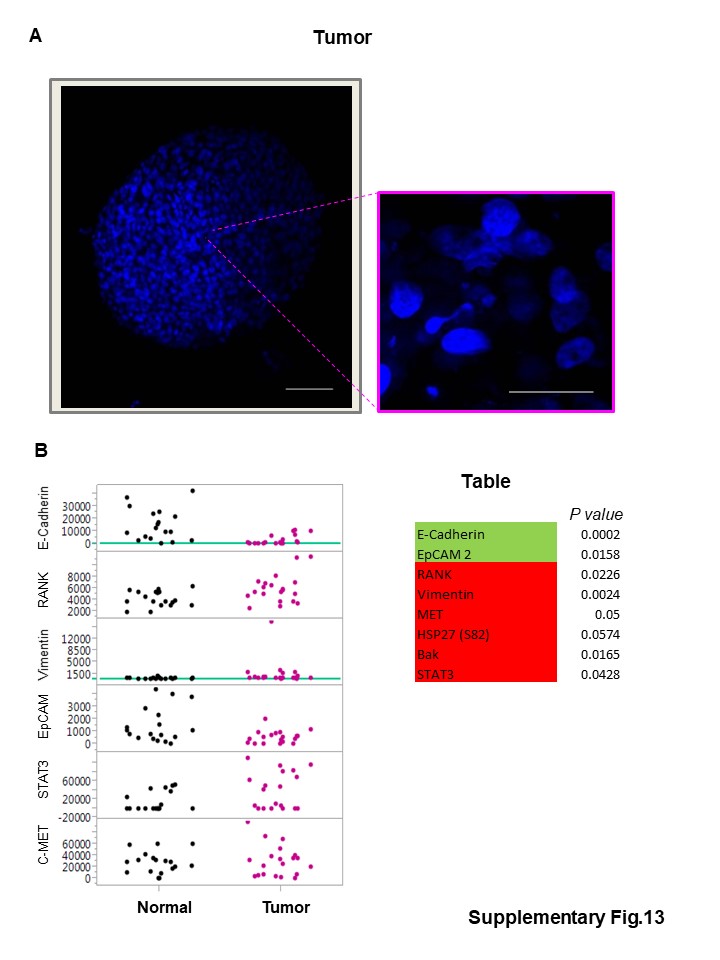

Supplement: Supplementary file 18 — Supplementary Fig.13 [file 41419_2019_1453_MOESM18_ESM.jpg]

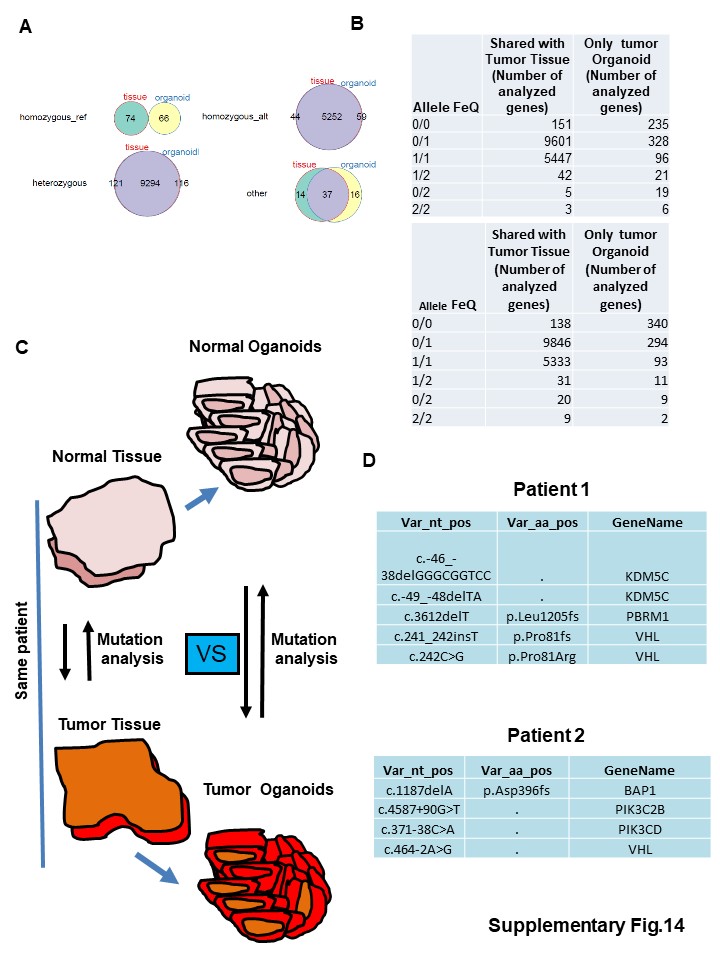

Supplement: Supplementary file 19 — Supplementary Fig.14 [file 41419_2019_1453_MOESM19_ESM.jpg]

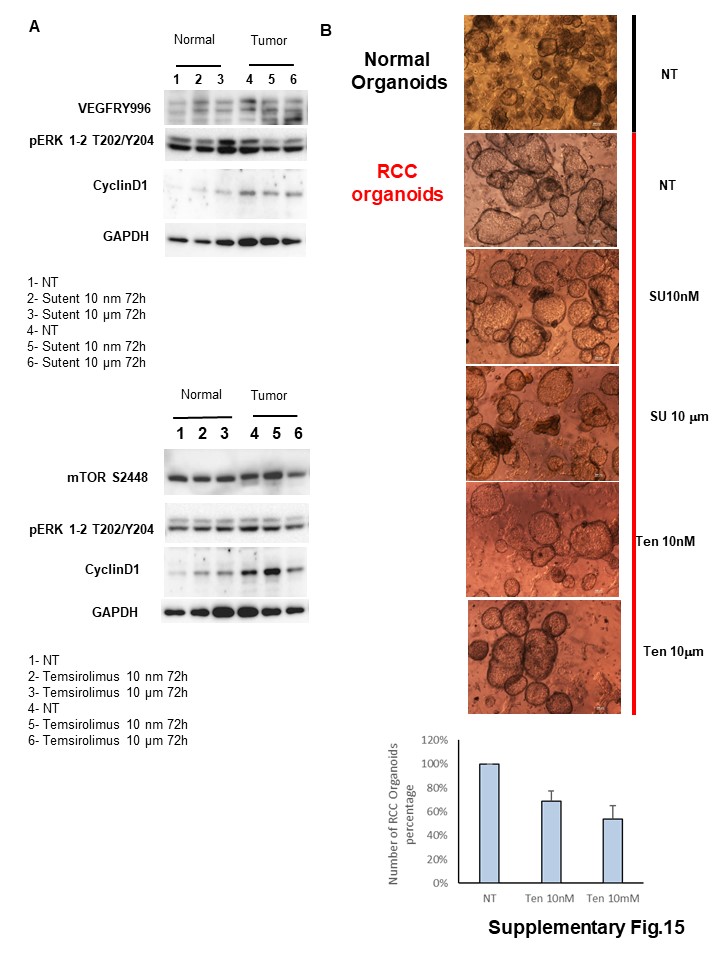

Supplement: Supplementary file 20 — Supplementary Fig.15 [file 41419_2019_1453_MOESM20_ESM.jpg]

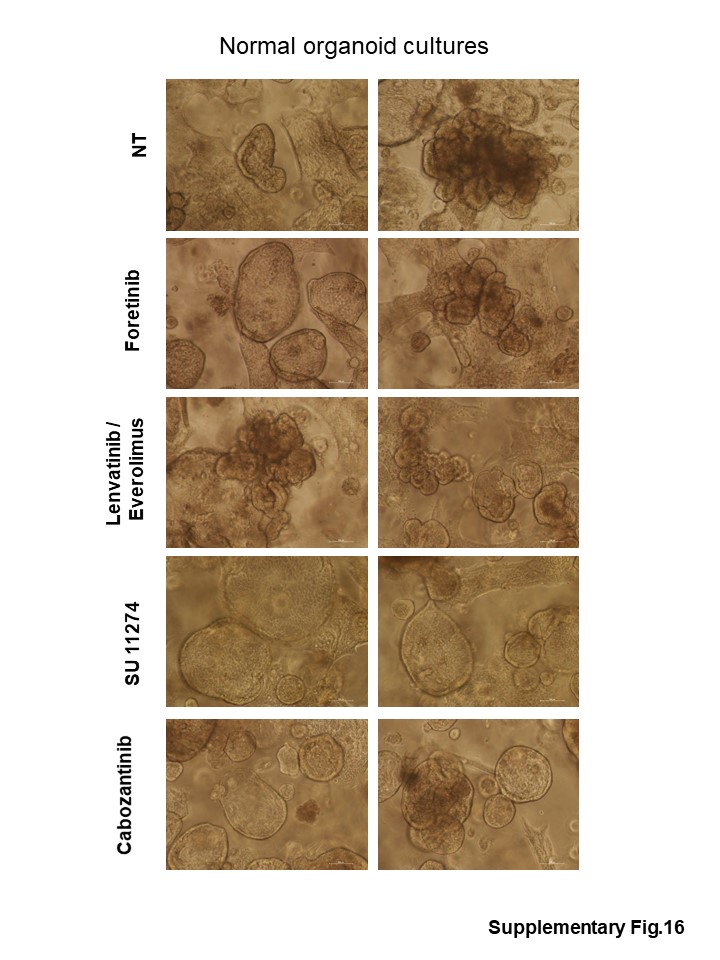

Supplement: Supplementary file 21 — Supplementary Fig.16 [file 41419_2019_1453_MOESM21_ESM.jpg]

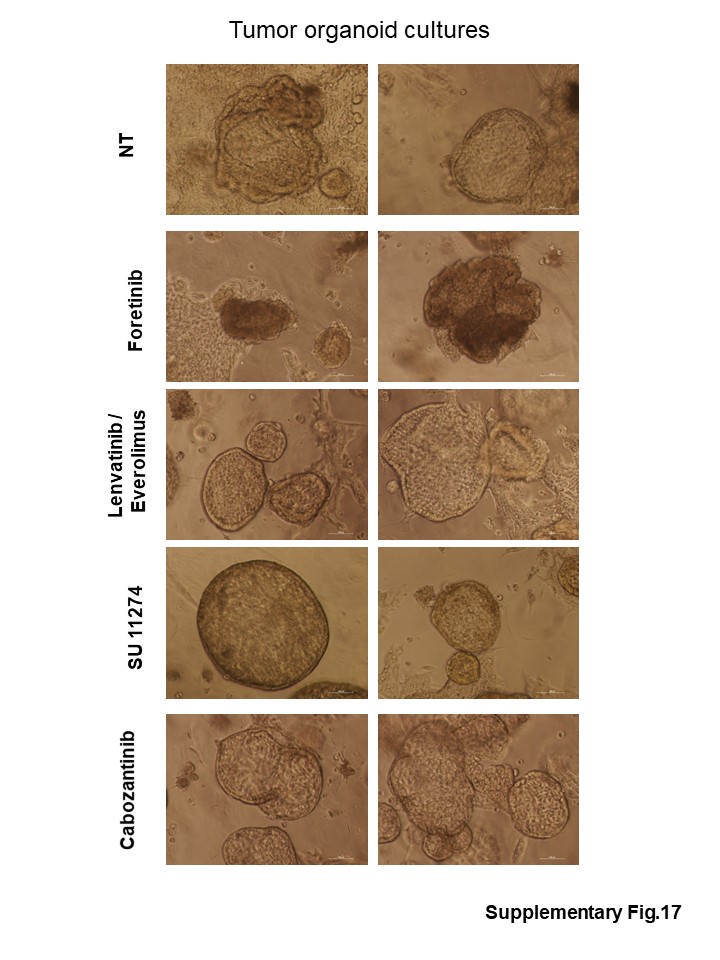

Supplement: Supplementary file 22 — Supplementary Fig.17 [file 41419_2019_1453_MOESM22_ESM.jpg]

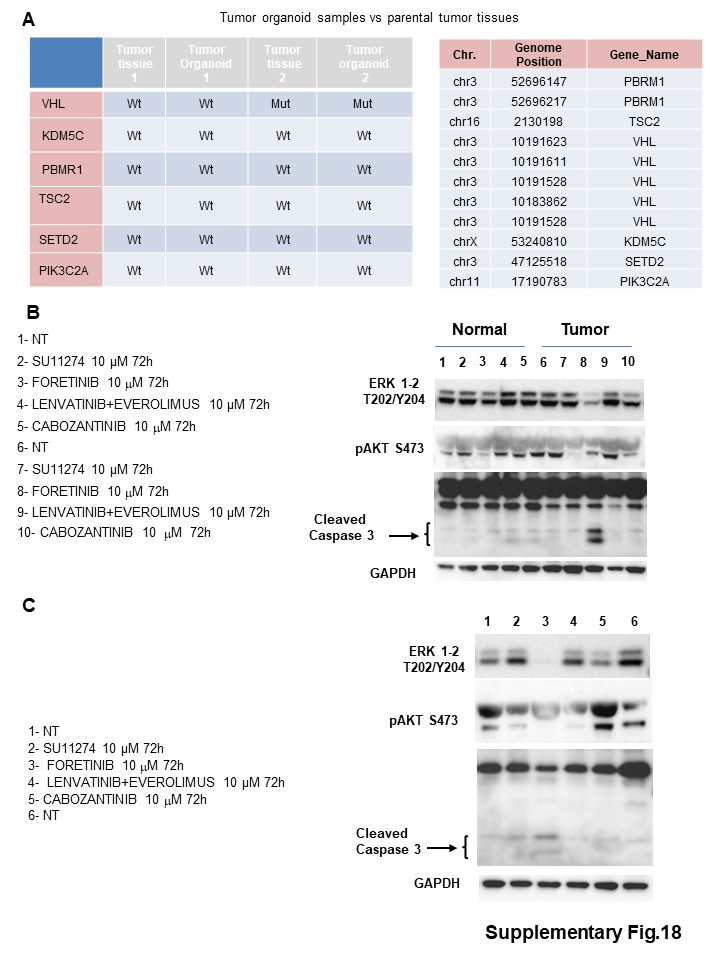

Supplement: Supplementary file 23 — Supplementary Fig.18 [file 41419_2019_1453_MOESM23_ESM.jpg]

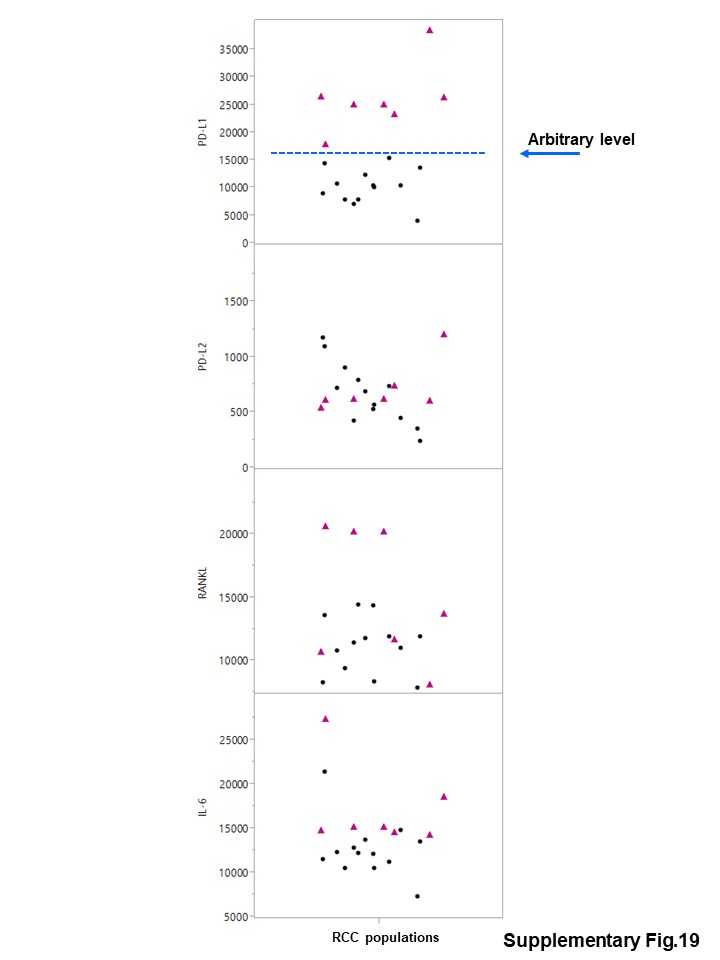

Supplement: Supplementary file 24 — Supplementary Fig.19 [file 41419_2019_1453_MOESM24_ESM.jpg]

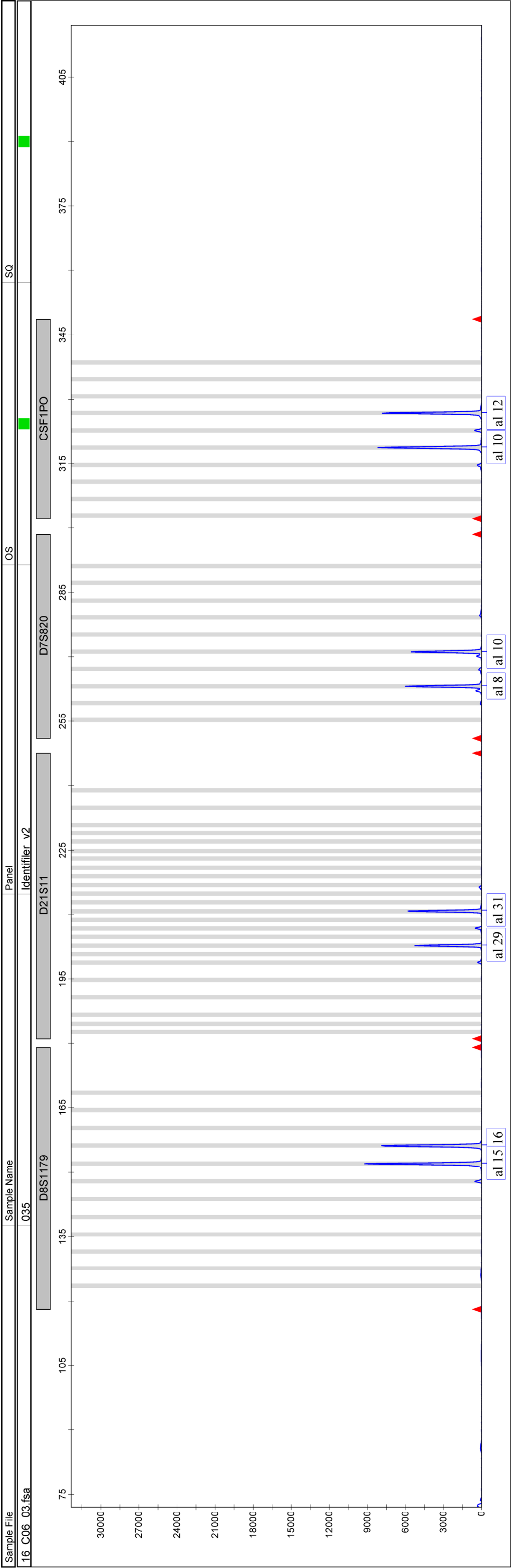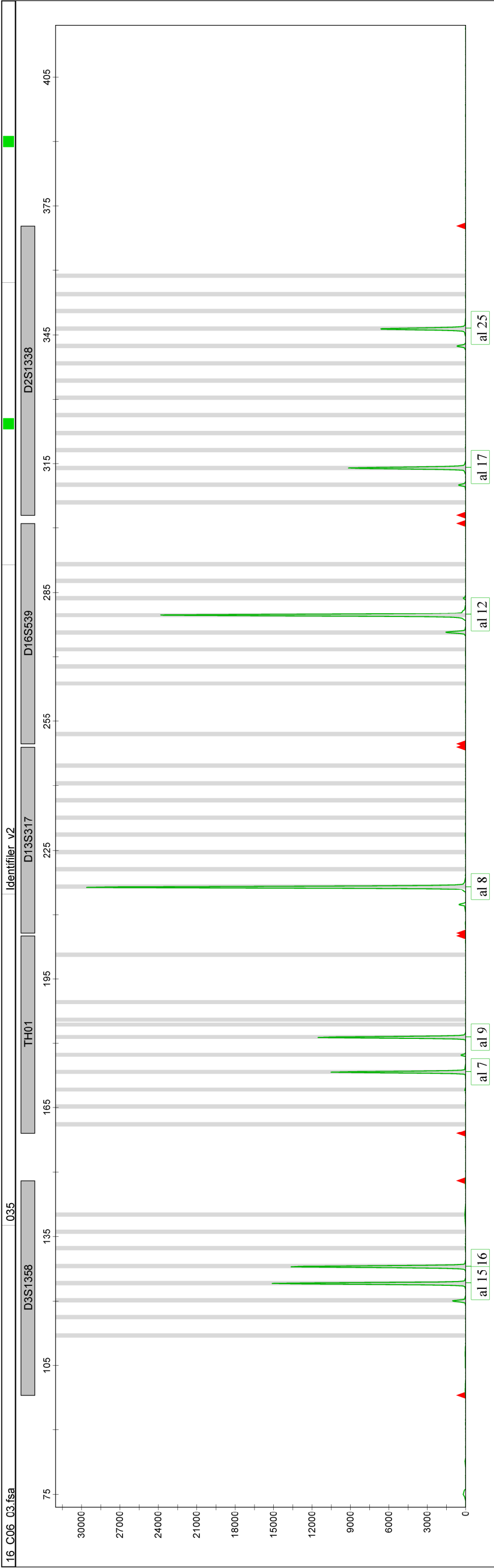

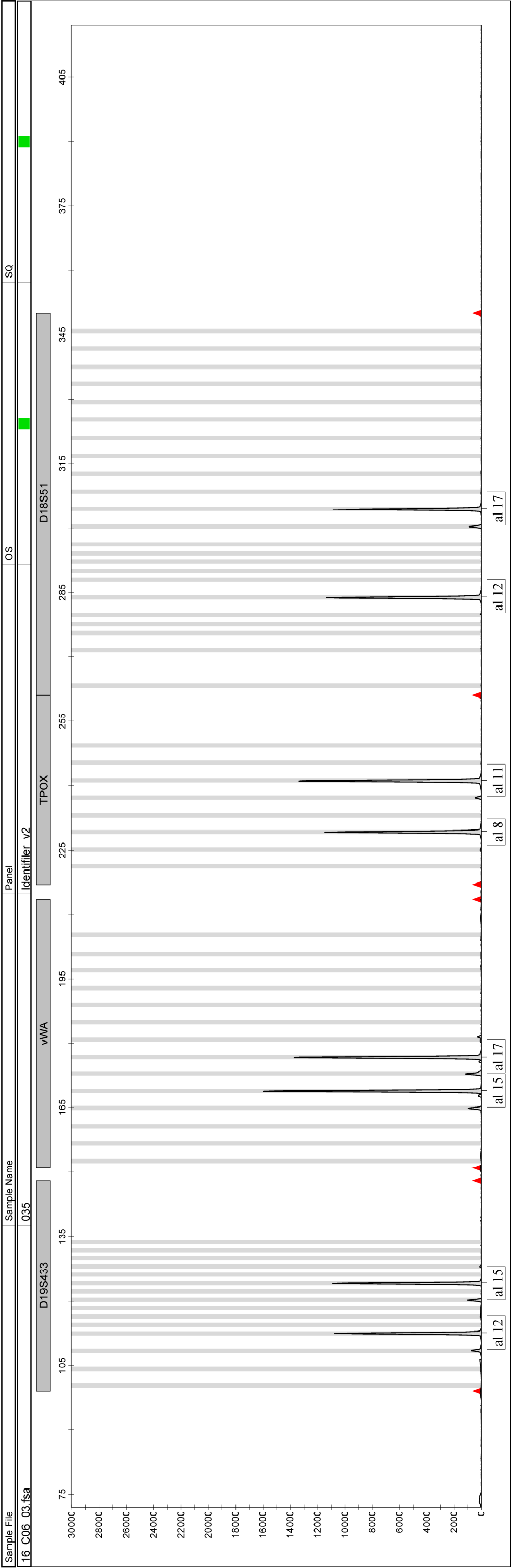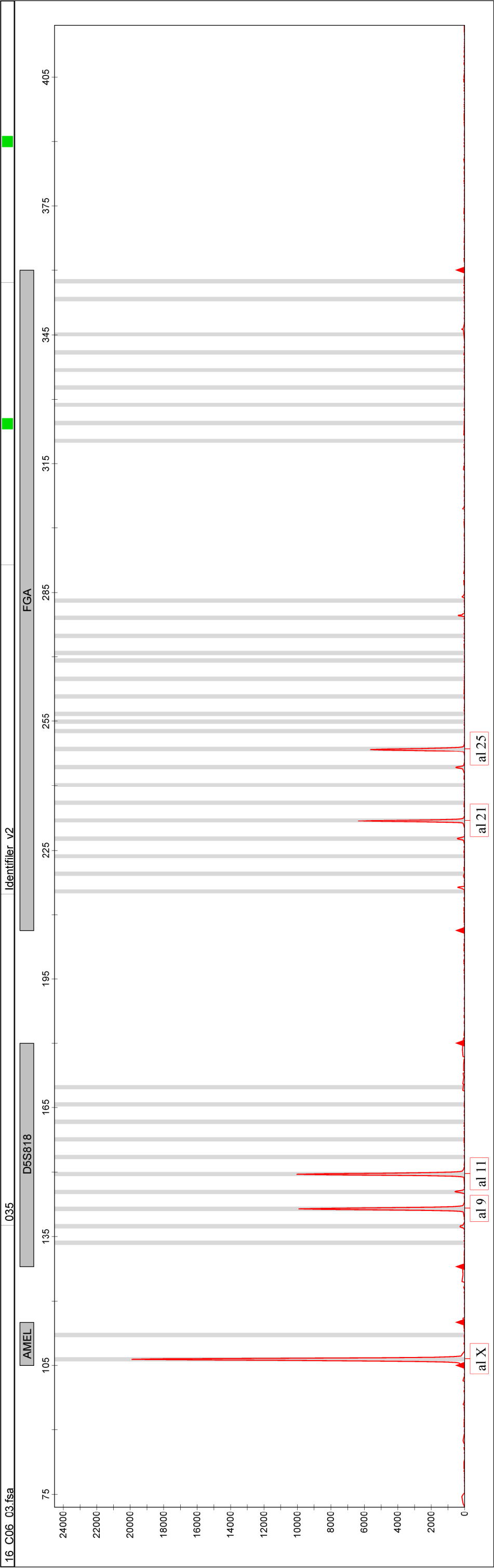

| Sample File   | Sample Name | Panel         | OS | SQ |
|---------------|-------------|---------------|----|----|
| 16_C06_03.fsa | 035         | Identifier v2 |    |    |

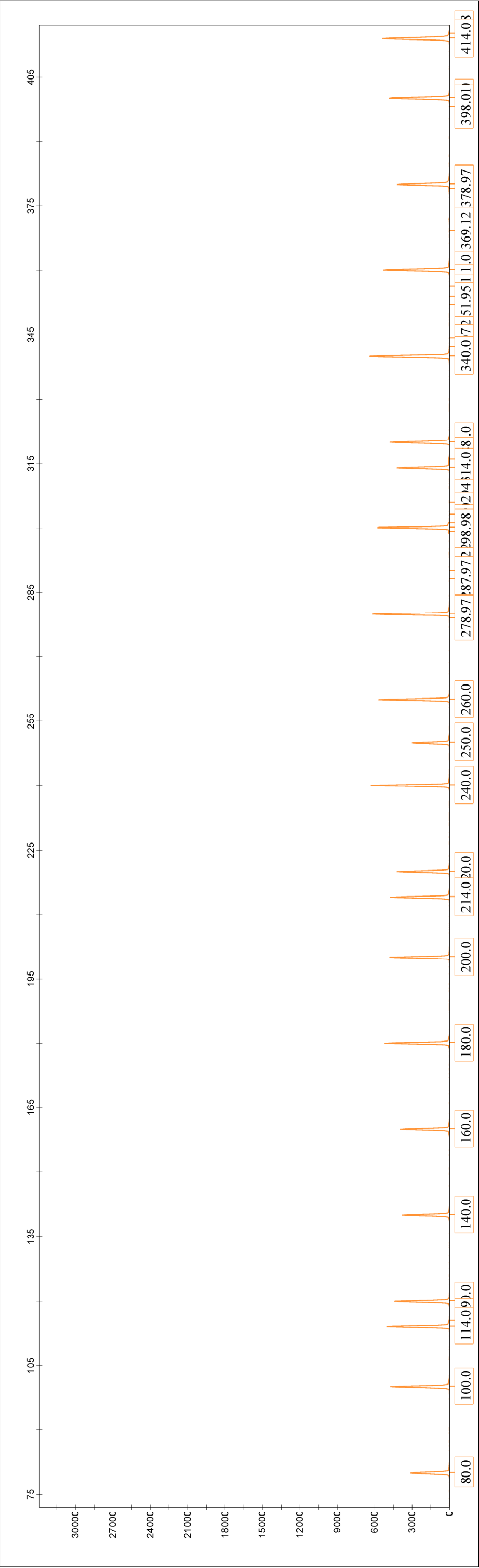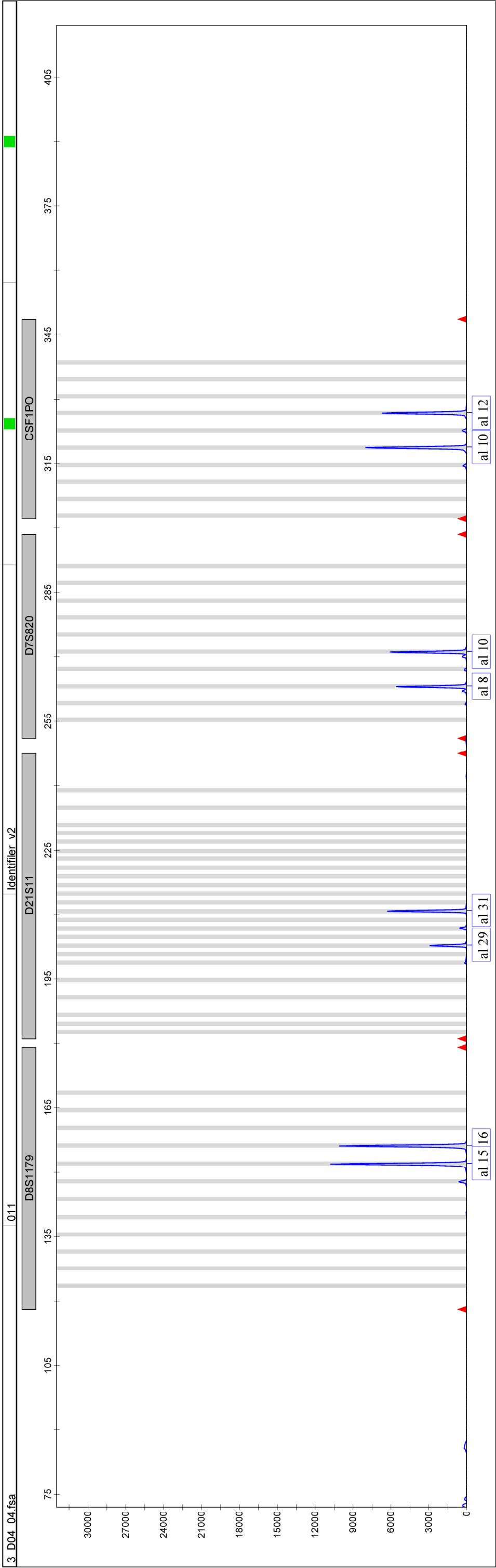

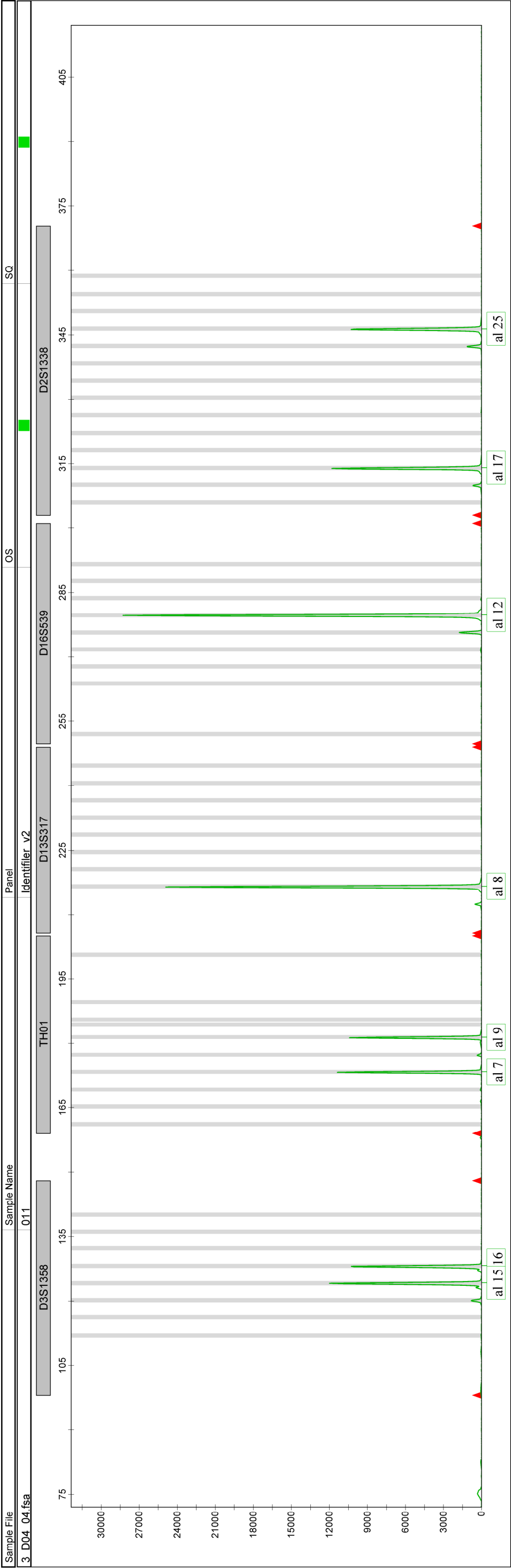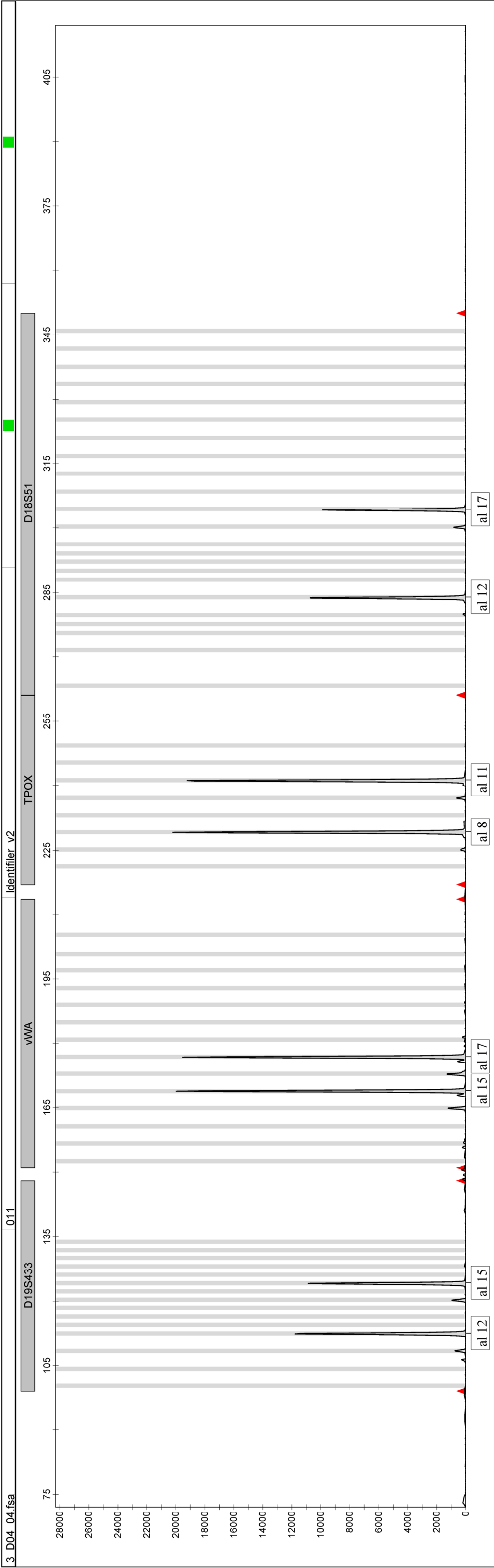

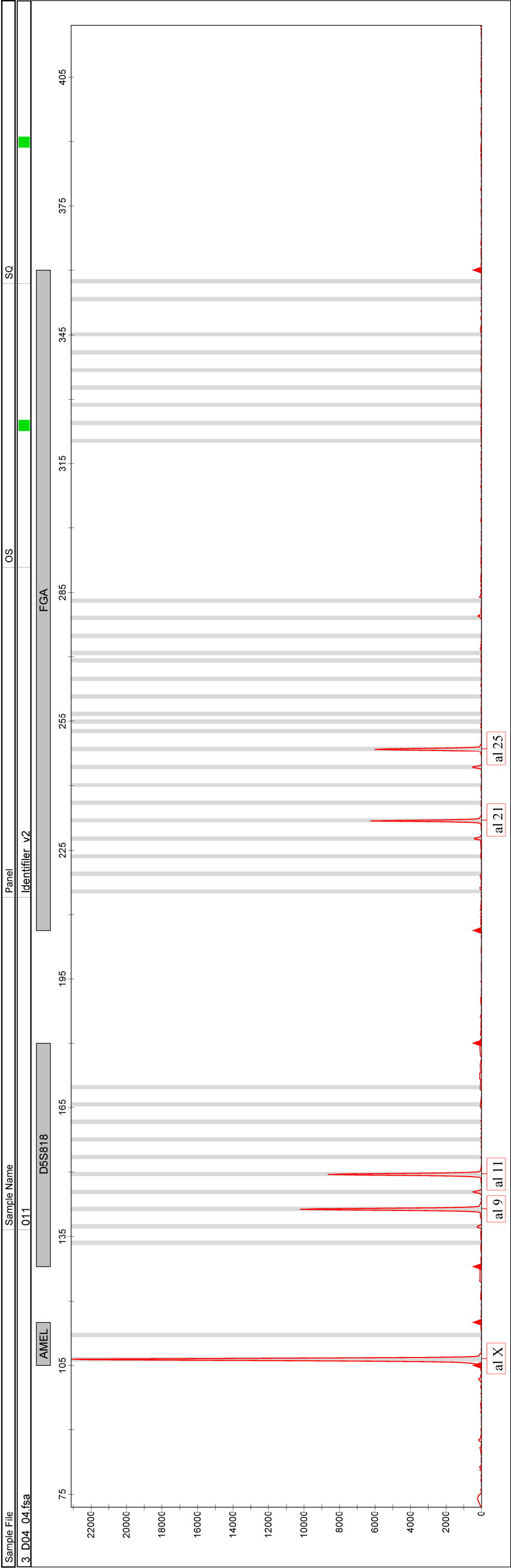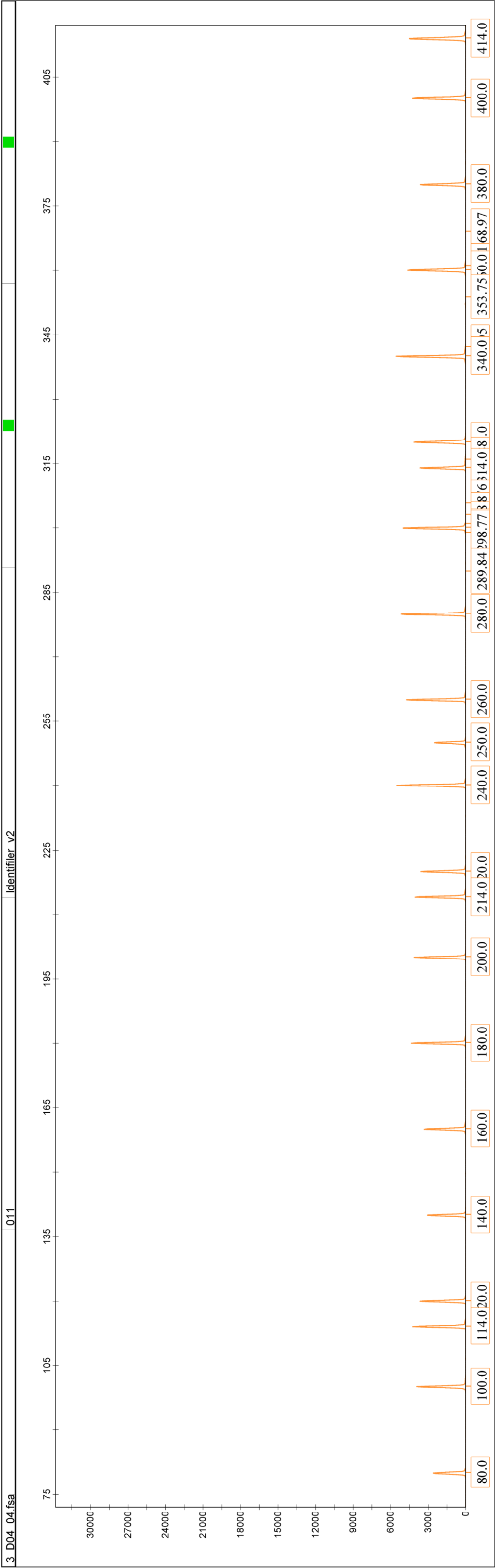

Supplement: Supplementary file 25 — Supplementary File 1 [file 41419_2019_1453_MOESM25_ESM.pdf]

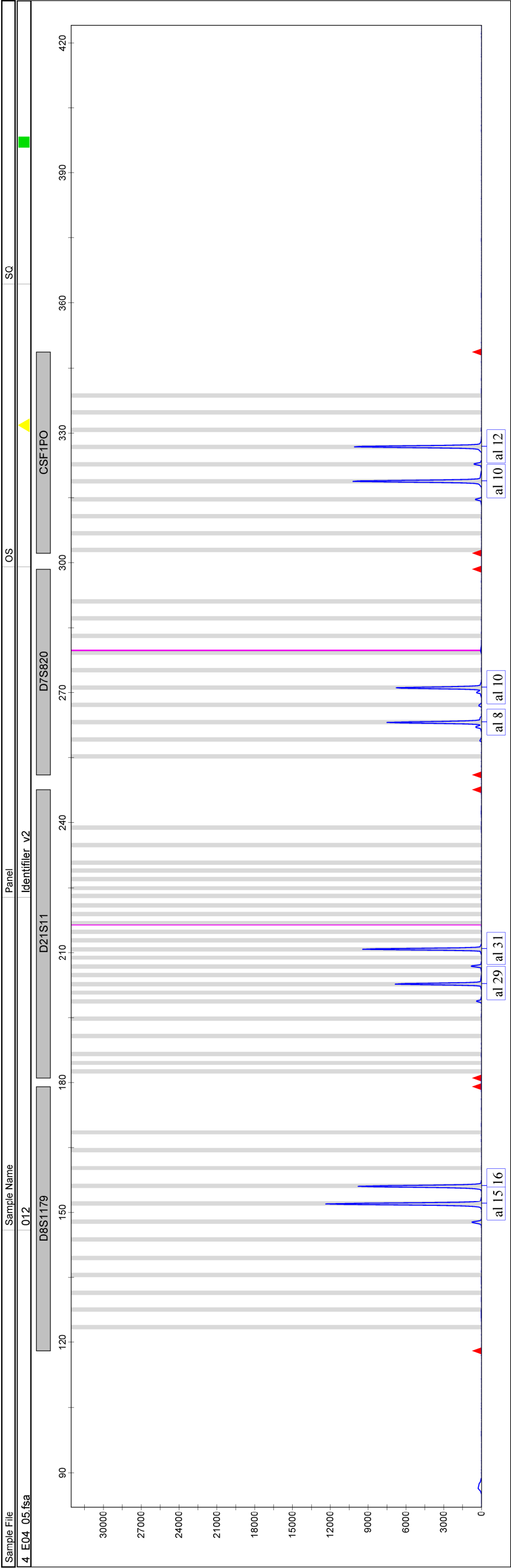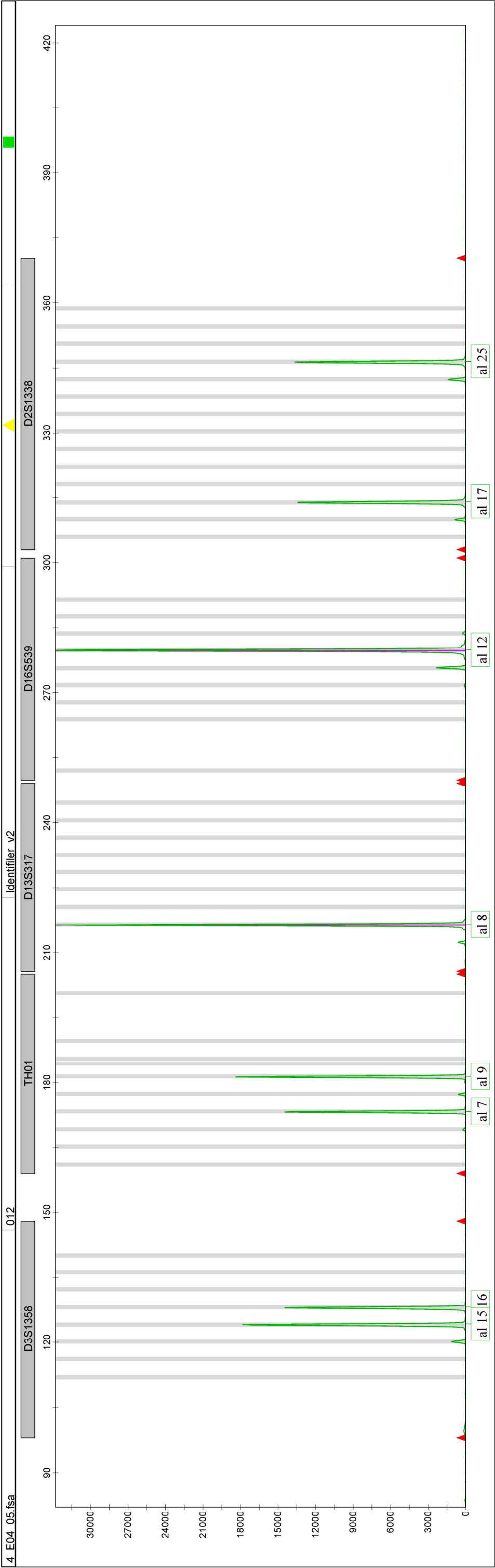

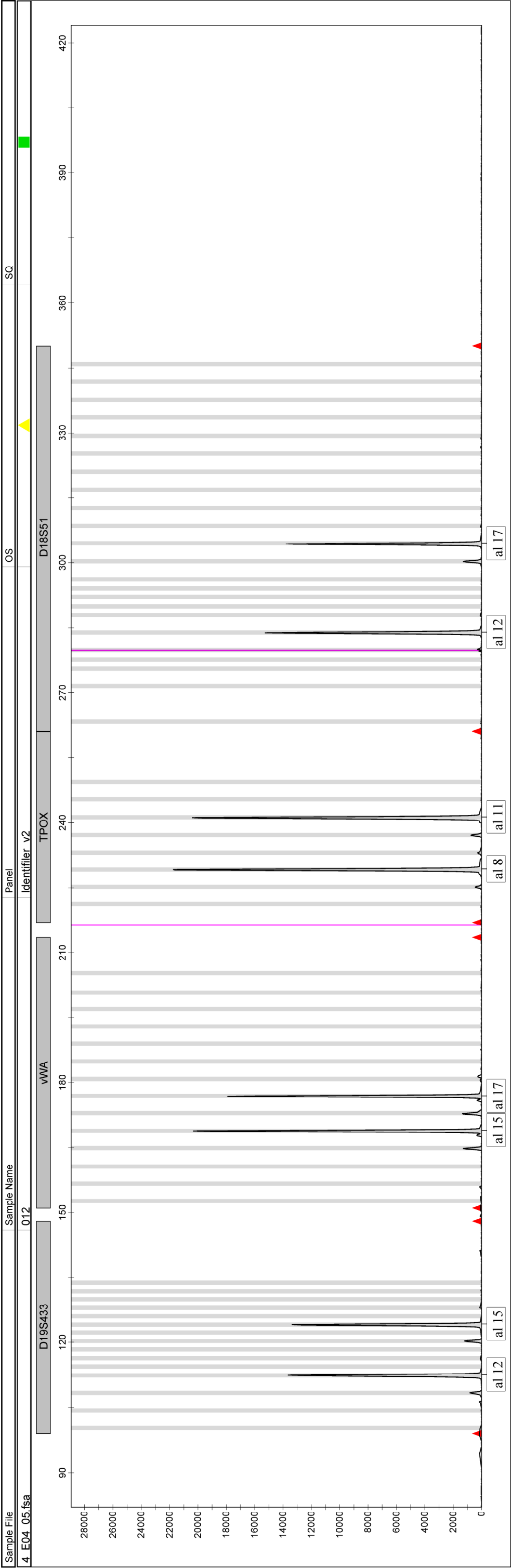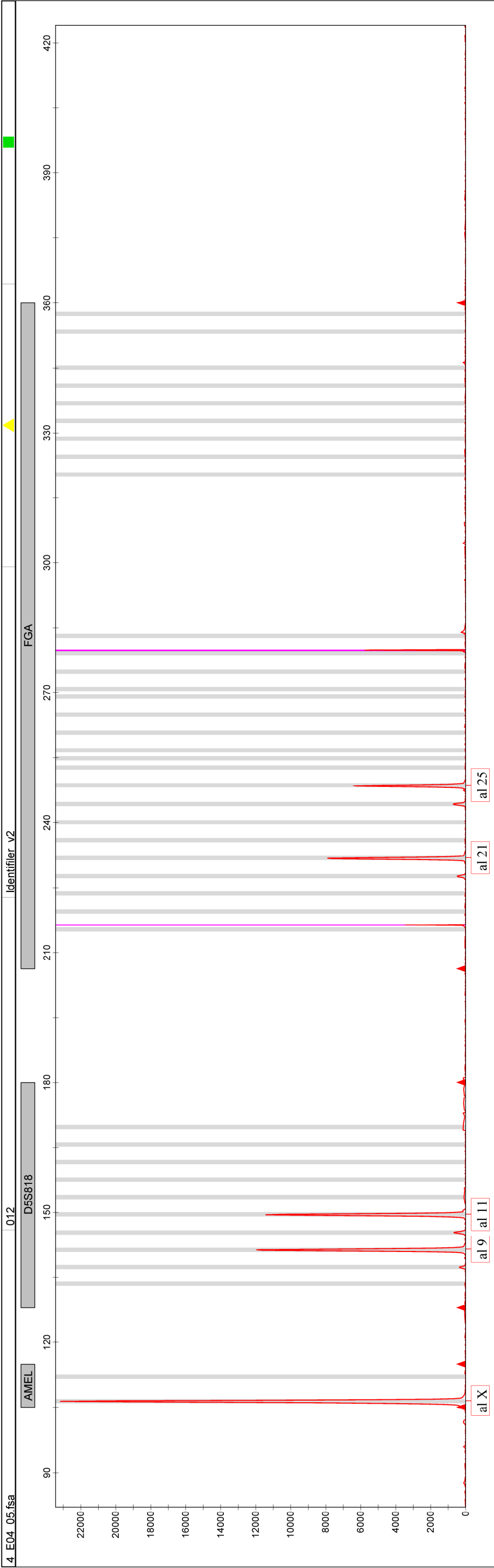

| Sample File  | Sample Name | Panel         | OS | SQ |
|--------------|-------------|---------------|----|----|
| 4_E04_05.fsa | 012         | Identifier v2 |    |    |

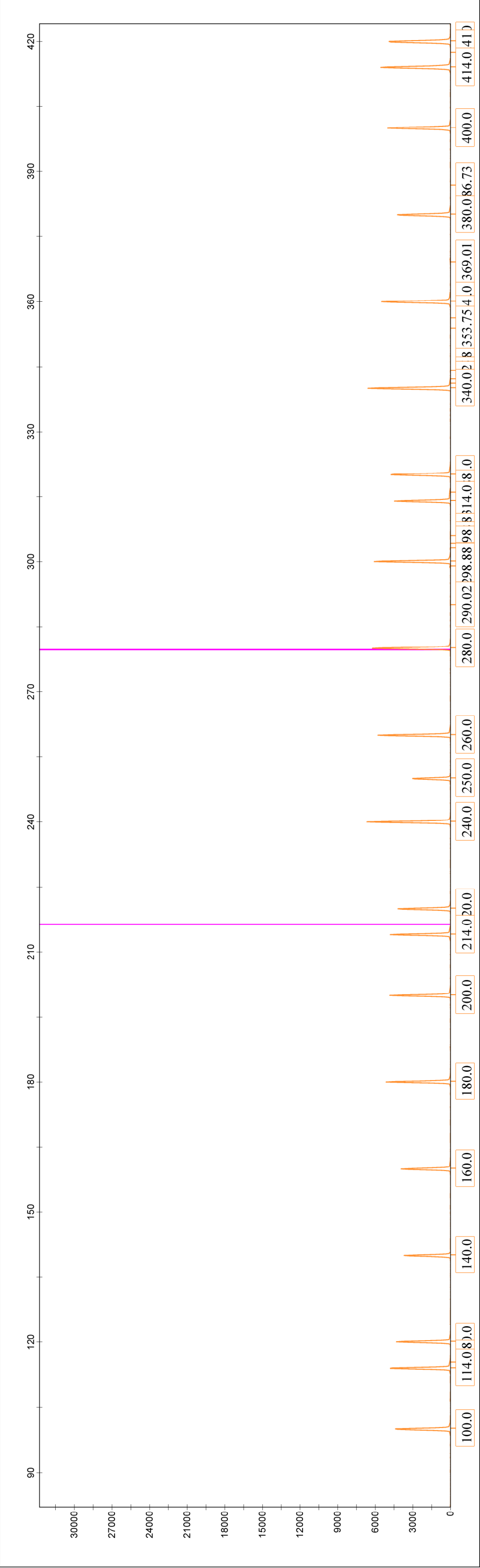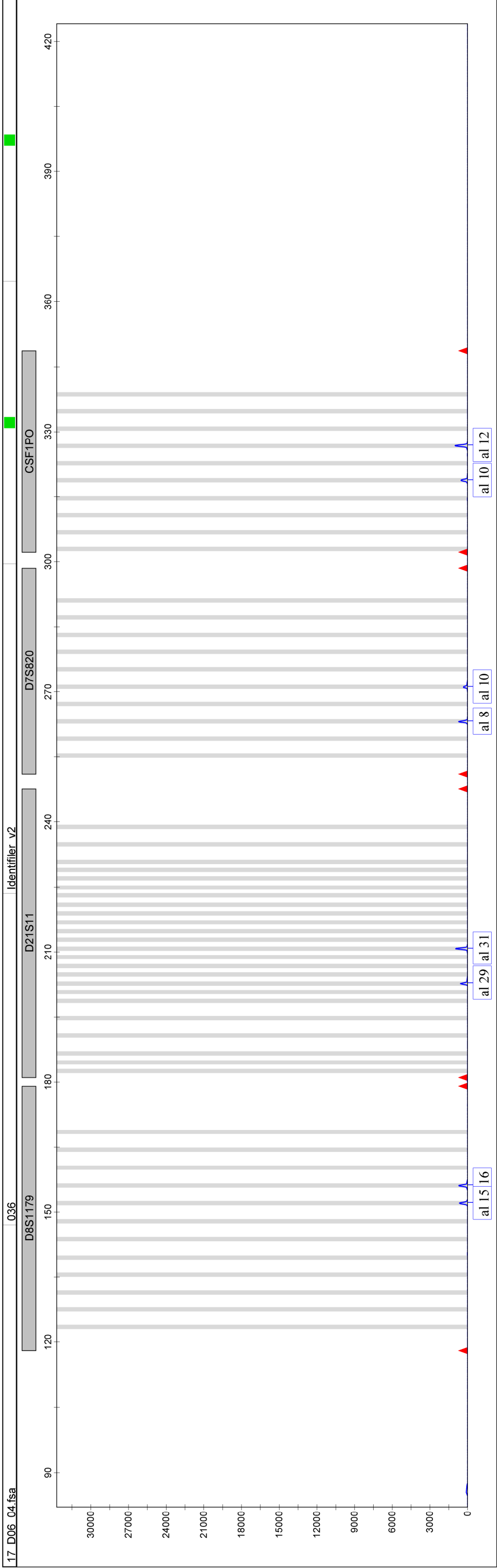

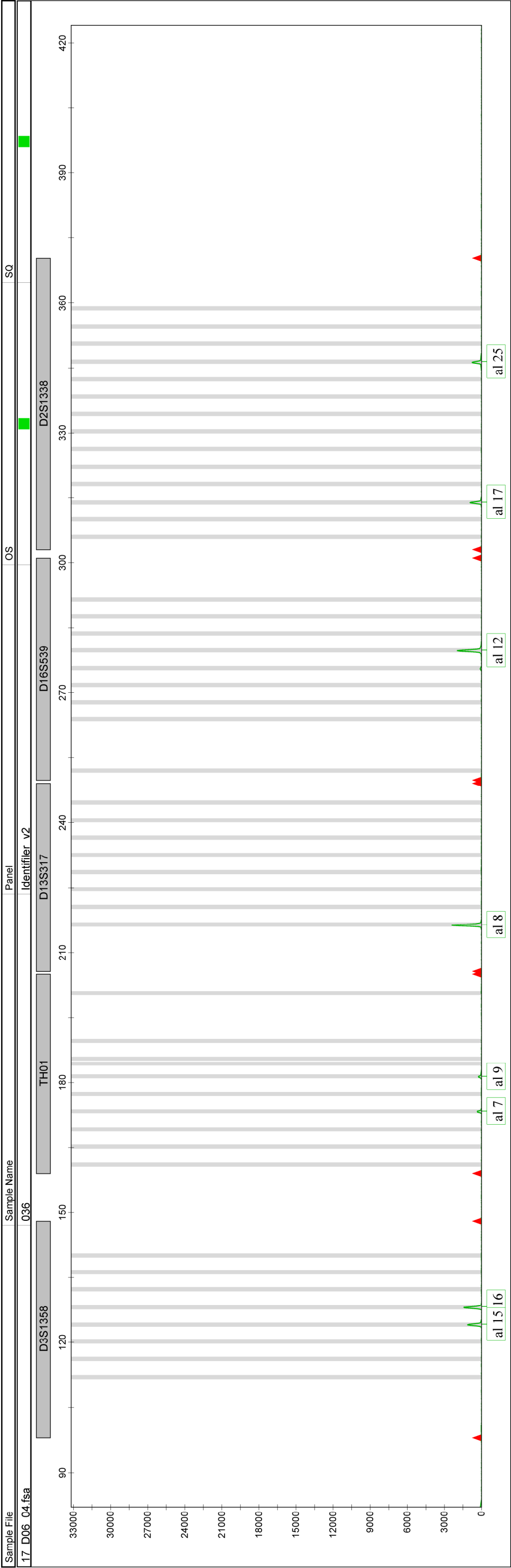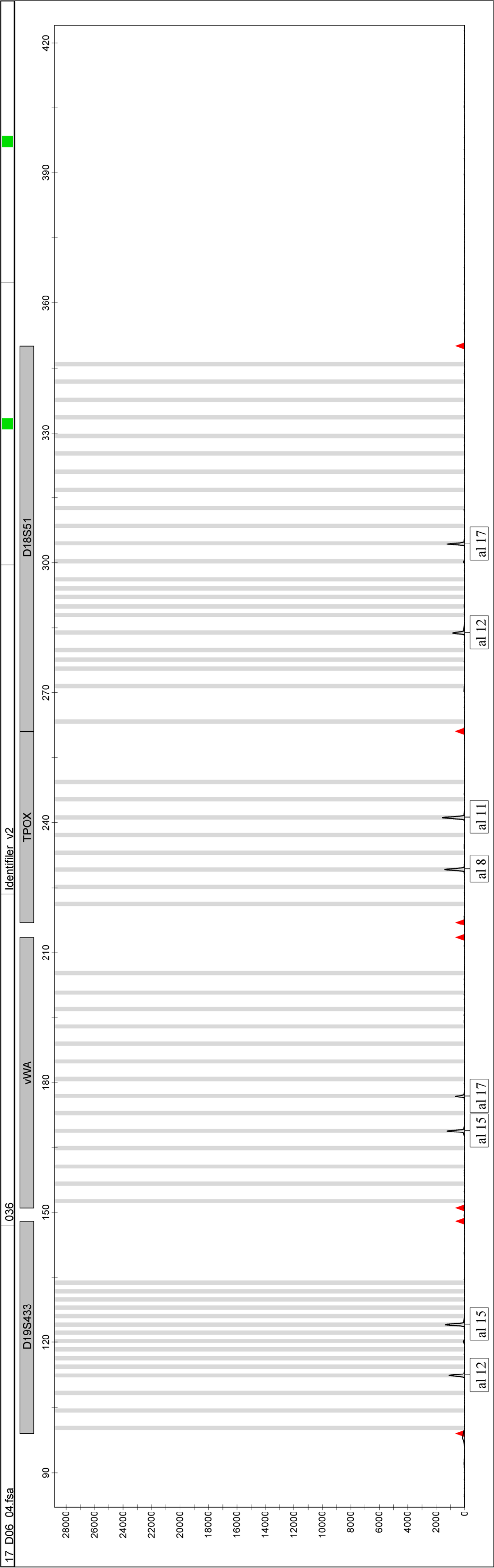

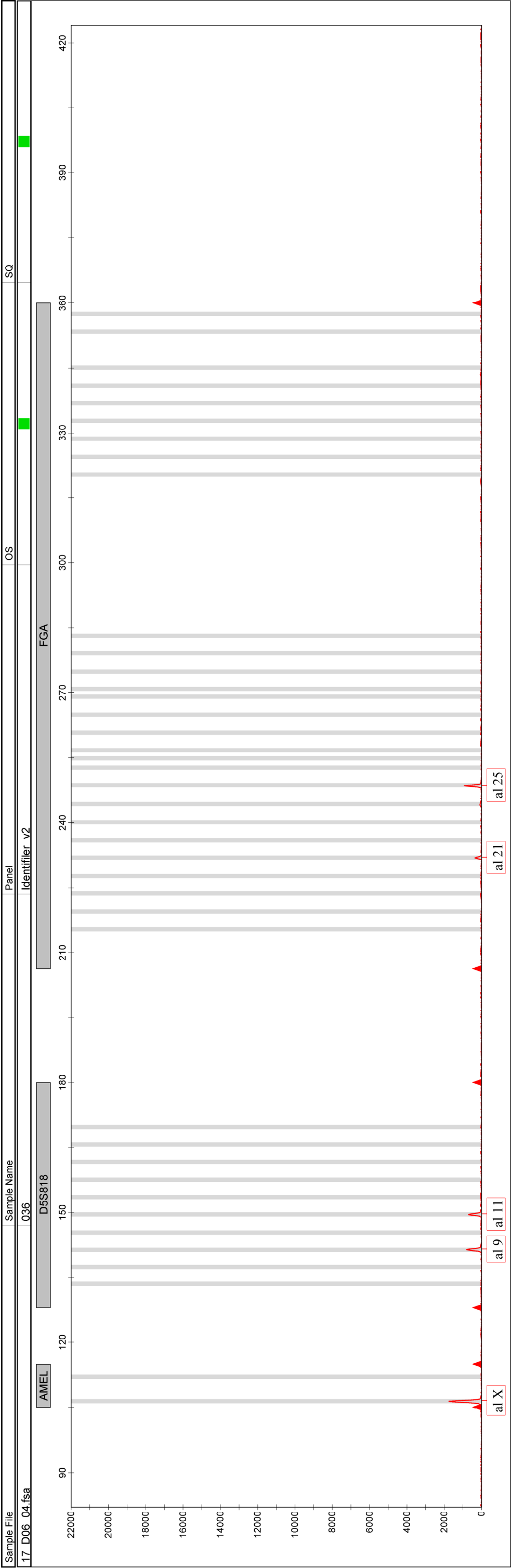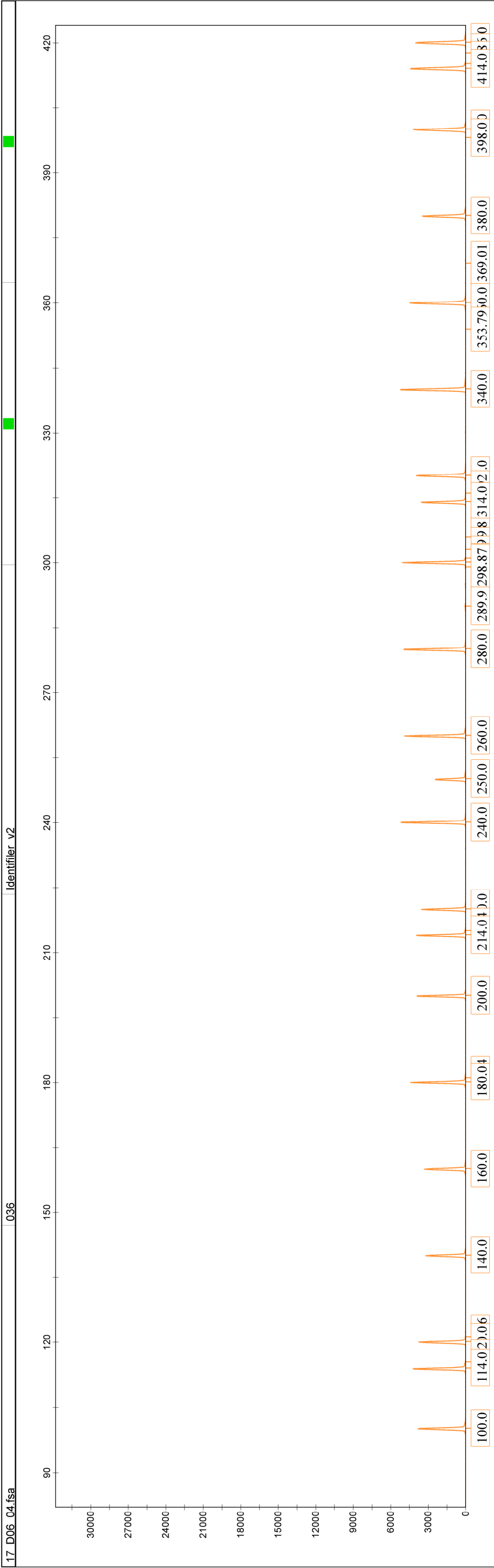

Supplement: Supplementary file 26 — Supplementary File 2 [file 41419_2019_1453_MOESM26_ESM.pdf]
